# Energy-Aware AI-Enabled Intelligent Sensing Systems for IoT and Edge–Cloud Architectures

**DOI:** 10.3390/s26216927

**Published:** 2026-11-02

**Authors:** Zhanar Mukash, Dina Bakranova, Abay Serikkanov, Nurlan Bakranov, Binara Imankulova

**Affiliations:** 1 School of Education and Humanities, SDU University, Kaskelen 040900, Kazakhstan; 2 School of Information Technologies and Applied Mathematics, SDU University, Kaskelen 040900, Kazakhstan; 3 Institute of Physics and Technology, Satbayev University, Almaty 050032, Kazakhstan; 4 National Academy of Sciences of the Republic of Kazakhstan Under the President of the Republic of Kazakhstan, Almaty 050000, Kazakhstan; 5 Faculty of Intelligent and Engineering Systems, Almaty Technological University, Almaty 050061, Kazakhstan; 6 Research Group AltAir Nanolab, Almaty 050043, Kazakhstan

**Keywords:** Internet of Things (IoT), edge–cloud computing, AI-enabled intelligent sensing, resource-aware artificial intelligence, distributed sensing architectures, energy-aware communication systems

## Abstract

AI-enabled intelligent sensing systems are increasingly used in Internet of Things (IoT) applications, edge–cloud environments, industrial automation, and cyber-physical systems. Their design, however, requires simultaneous consideration of sensing performance, computational demand, communication requirements, and limited energy resources. Much of the existing literature addresses these aspects separately, although their interactions often determine the performance and autonomy of the complete sensing platform. This review examines energy-aware AI-enabled sensing systems from a system-level perspective, with particular attention to the trade-offs that arise under resource-constrained operating conditions. Representative studies spanning intelligent sensing, embedded intelligence, edge–cloud computing, wireless sensor networks, AIoT, and energy-aware system design are analyzed to identify common architectural principles and design constraints. Four recurring system-level trade-offs are considered, namely measurement accuracy versus energy consumption, latency versus energy savings, local computation versus communication load, and model fidelity versus computational efficiency. The review further develops a multidimensional taxonomy of energy-aware sensing architectures based on energy source, operational mode, processing model, and communication strategy. These elements are integrated into a common framework linking sensing performance, computational requirements, communication demand, and energy allocation. Particular attention is also given to hybrid and multimodal sensing, distributed intelligence, TinyML, hardware-aware model deployment, and adaptive resource allocation. Remaining challenges include scalability, operation under changing sensing conditions, cross-platform evaluation, trustworthy embedded AI, and the transition from laboratory demonstrations to long-term deployment. The resulting system-level perspective provides a basis for designing intelligent sensing platforms in which sensing, computation, communication, and energy resources are considered jointly rather than optimized in isolation.

## 1. Introduction

The rapid proliferation of sensor technologies has transformed modern monitoring and sensing systems across a wide range of application domains, including environmental monitoring, industrial automation, healthcare, smart infrastructure, and cyber-physical systems. Advances in sensing devices, embedded electronics, wireless communication, and artificial intelligence have enabled the deployment of increasingly autonomous sensing platforms capable of operating in dynamic and distributed environments [1,2,3,4,5]. As a result, sensor systems are no longer viewed as isolated measurement devices but as integrated intelligent platforms responsible for data acquisition, local processing, communication, and decision-making [6,7].

Despite these advances, energy availability remains one of the most critical constraints limiting the performance and long-term operation of intelligent sensing systems [8]. Many practical deployments rely on batteries, energy harvesting, or hybrid power sources that provide limited and environmentally dependent energy resources. Under such conditions, energy consumption directly influences sensing frequency, communication activity, computational complexity, system responsiveness, and operational lifetime [9,10,11]. Consequently, the design of modern sensor systems cannot be reduced to maximizing measurement accuracy alone. Instead, it requires balancing sensing performance with energy efficiency and resource utilization [12,13,14,15,16].

At the same time, the increasing integration of artificial intelligence has fundamentally changed the architecture of sensor systems. Traditional sensing platforms typically relied on centralized cloud-based processing, where large volumes of raw data were transmitted to remote servers for analysis [17,18]. While such approaches offer significant computational capabilities, they also introduce communication overhead, latency, and increased energy consumption [19,20,21]. Recent developments in edge computing, TinyML, and embedded machine learning have enabled a shift toward distributed intelligence, where data processing and decision-making are performed directly on sensor nodes or edge devices. This transition reduces communication requirements and improves responsiveness but introduces new challenges associated with computational load, memory limitations, and energy consumption [22,23,24,25,26,27].

The convergence of sensing, communication, embedded intelligence, and energy management has created a new class of energy-aware intelligent sensor systems [28,29]. In these systems, overall performance emerges from the interaction of multiple interconnected subsystems rather than from the capabilities of individual components. Improvements in sensing quality may require additional computational resources. Reducing communication overhead through local inference may increase energy consumption associated with processing. Increasing model complexity may improve prediction accuracy while simultaneously reducing system autonomy [30,31]. These interactions create a series of fundamental trade-offs that govern system behavior under resource-constrained operating conditions. Although extensive research has been conducted on low-power sensing, wireless sensor networks, edge artificial intelligence, TinyML, and energy-aware communication protocols, these topics are frequently investigated as separate research directions. Existing studies often focus on individual performance metrics such as sensing accuracy, network lifetime, latency, throughput, or computational efficiency [32,33,34,35,36]. As a result, relatively limited attention has been devoted to understanding how these factors interact at the system level and collectively determine the performance of intelligent sensing platforms.

Recent developments further highlight the need for such an integrated perspective. The emergence of edge AI architectures, energy-harvesting sensor nodes, adaptive communication protocols, and resource-efficient machine learning models has shifted research attention from component-level optimization toward cross-layer system design [37].

In this context, achieving high performance requires coordinated optimization of sensing, computation, communication, and energy allocation rather than independent improvement of individual subsystems. Consequently, there is a growing need for conceptual frameworks that can explain these interactions and support the development of adaptive, scalable, and energy-efficient sensing architectures.

This review addresses this need by examining energy-aware AI-enabled sensor systems from a system-level perspective. Accordingly, conventional topics such as energy harvesting, routing, clustering, communication, and sensor design are considered only where they influence the resources available for embedded or distributed intelligence. These mechanisms determine the energy budget, data availability, communication cost, and operating conditions under which AI-enabled sensing can be deployed. Rather than focusing on specific sensing materials, individual devices, or isolated algorithms, the paper investigates how sensing, computation, communication, and energy management interact within intelligent sensing platforms operating under resource constraints. The analysis synthesizes representative research across edge intelligence, embedded machine learning, wireless communication, and energy-aware system design to identify common architectural principles and performance limitations.

Based on this synthesis, four fundamental system-level trade-offs are identified for intelligent sensing platforms, including measurement accuracy versus energy consumption, latency versus energy savings, local computation versus communication load, and model fidelity versus computational efficiency. Building on these trade-offs, this review develops a multidimensional taxonomy of energy-aware sensing architectures based on energy source, operational mode, processing model, and communication strategy. A unified conceptual framework is further established to relate sensing performance, computational requirements, communication demand, and energy allocation within a common design space. Together, these elements provide a basis for examining current limitations and research directions toward adaptive, scalable, and resource-efficient intelligent sensing systems.

### Literature Search and Selection

A structured narrative review methodology was adopted to identify and synthesize relevant literature on energy-aware sensor systems, edge intelligence, and system-level design trade-offs. Searches were conducted in Scopus and Web of Science (WoS), with the final search performed in June 2026. The search covered publications from 2008 to June 2026 and focused on the interrelationships among sensing, data acquisition, edge computing, wireless communication, cloud analytics, and energy management.

The search strategy combined keywords and related expressions covering the principal themes of the review. The search terms included “energy-aware sensing,” “low-power sensor systems,” “edge computing,” “embedded artificial intelligence,” “TinyML,” “wireless sensor networks,” “energy harvesting,” “resource-aware computing,” and “system-level trade-offs.” Boolean operators were used to combine related terms and identify publications addressing one or more of the review’s principal research areas. The searches were limited to the specified publication period.

Records retrieved from the two databases were consolidated, and duplicate records were identified and removed before screening. The remaining records were assessed for relevance based on their titles and abstracts. Publications potentially relevant to the review were then evaluated against the full-text eligibility criteria. Studies were included if they (1) were published between 2008 and June 2026; (2) addressed at least one of the principal research areas covered by the review; and (3) provided conceptual, architectural, experimental, or quantitative evidence relevant to the relationships among sensing, computation, communication, and energy management. Records were excluded if their primary focus fell outside the scope of the review or if they did not contribute directly to the analysis of the system-level trade-offs under consideration.

Given the interdisciplinary and evolving nature of the field, the selection process also involved iterative examination of relevant references to identify additional publications that contributed to the conceptual framework and critical discussion. The literature search initially identified 482 records from the selected sources. Following the removal of 95 duplicate records, 387 publications remained for title and abstract screening. Based on their relevance to the scope and objectives of this review, 180 articles were selected for full-text assessment. After a detailed evaluation of their methodological relevance, technical content, and contribution to the research topic. The final bibliography comprises 131 references, including journal articles, conference papers, and a relevant book chapter. The number of references in the final bibliography reflects the sources cited throughout the manuscript, whereas the number of records retrieved, screened, and included in the core literature synthesis is reported separately in the selection summary.

## 2. System-Level Perspective on Intelligent Sensing Systems

The development of modern sensor systems is increasingly moving beyond the traditional paradigm in which sensing, data processing, and communication modules are designed independently. In practical applications, particularly in distributed and autonomous environments, these components operate as tightly coupled subsystems, with overall performance determined not by the characteristics of individual elements alone, but by their interactions under resource constraints. This system-level perspective is consistent with recent interdisciplinary studies emphasizing that effective sensor architectures require coordinated integration of sensing, communication, computation, and decision-making rather than isolated optimization of individual components [38].

Table 1 shows comparison of positioning the present review against representative surveys on energy-aware WSNs, Internet of Things (IoT), Edge AI, and TinyML.

The comparison in Table 1 reveals three main limitations in the existing survey literature. First, surveys on energy-efficient and sustainable wireless sensor networks [39,40,41] primarily examine energy management, communication, and network lifetime, with less emphasis on the computational requirements of embedded AI. Second, reviews of Edge AI and TinyML [42,43,44] focus on processing architectures, model optimization, and hardware constraints, while providing less integrated consideration of sensing operation and energy-source characteristics. Third, these research directions are generally examined through different taxonomies and evaluation criteria, making it difficult to analyze how architectural choices affect multiple system resources simultaneously.

The present review addresses these limitations by organizing energy-aware intelligent sensing architectures along four complementary dimensions: energy source, operational mode, processing model, and communication strategy. Unlike classifications focused on individual technologies, the proposed taxonomy allows these dimensions to be examined together and related to four recurring system-level trade-offs: measurement accuracy versus energy consumption, latency versus energy savings, local computation versus communication load, and model fidelity versus computational efficiency. The main contribution is therefore a common analytical structure for synthesizing existing findings, identifying interactions among architectural decisions, and highlighting research gaps that are less apparent when sensing, computation, communication, and energy management are considered separately.

The implications of this interdependence are particularly evident in distributed sensing networks, where sensing duration, detection thresholds, node participation, and data transmission strategies are closely connected. In cognitive radio and related adaptive sensing systems, for example, increasing sensing time can improve detection reliability while reducing the time available for data transmission and, consequently, achievable throughput. Similarly, increasing the number of participating sensing nodes may enhance detection performance, but also increases energy consumption and communication overhead [45]. These relationships show that improvements in sensing performance can impose costs elsewhere in the system and therefore require joint consideration of sensing, communication, and energy use.

Energy availability imposes an additional constraint on this balance. Battery-powered sensor nodes operate with a limited energy budget, whereas energy-harvesting nodes are often subject to intermittent and time-varying energy availability. In both cases, energy management directly influences operational lifetime, availability, and reliability [46]. Network-level approaches such as dynamic resource allocation, duty-cycle control, and adaptive scheduling have therefore become important mechanisms for matching system activity to available energy. In wireless rechargeable sensor networks, for instance, charging schedules can prioritize nodes according to residual energy, consumption rate, and functional role, thereby improving network sustainability [47]. Such strategies can extend operation, but they do not remove the need to allocate energy efficiently among competing functions.

At the node level, the available energy budget must be divided among sensing, computation, memory access, and communication. The signal acquisition and processing chain can therefore be viewed as a sequence of interconnected stages in which signal-to-noise ratio, resolution, sampling requirements, processing complexity, and power consumption are mutually dependent [48]. Higher sampling rates, greater measurement resolution, or multimodal sensing can increase the amount and quality of acquired information, but they also increase data-processing and transmission requirements. Local computation introduces a related trade-off. Edge AI and TinyML can process data directly at the sensor node, enabling local inference, event detection, and data reduction before transmission [49]. This can decrease communication traffic and latency, but additional computation and memory access consume energy. The optimal operating point consequently depends on whether the energy saved by reducing communication exceeds the additional cost of local processing.

Edge AI and TinyML can reduce communication by processing data locally, although computation and memory access also consume energy. The benefit depends on whether the energy saved through reduced communication exceeds the additional processing cost. Measurements reported in [50] illustrate this trade-off for an Arduino Nano 33 BLE operating at 3.3 V. Local inference consumed approximately 9.1 mJ, while transmitting an image and receiving the remote inference result required about 111.8 mJ. When transmission of the locally generated result was included, the local processing and communication energy increased to approximately 70.5 mJ, still about 37% lower than the remote inference path.

These values were calculated from the reported average current and execution time of each operation using *E* = *U* × *I* × *t*. The comparison covers energy consumed by the sensor device during inference and communication. Image acquisition and result indication, which are common to both paths, are excluded, as are the energy costs of gateway and cloud processing. The results therefore describe device-side energy consumption under the conditions reported in [50], rather than the total energy consumption of the complete system.

The break-even condition for local inference can be expressed as:

*E_inf_* + *E_ts_*_,*res*_ < *E_tx_*_,*img*_ + *E_rx_*_,*res*_

where *E_inf_* is the energy required for local inference, *E_tx_*_,*res*_ is the energy used to transmit the locally generated result, *E_tx_*_,*img*_ is the energy required to transmit the original image, and *E_rx_*_,*res*_ is the energy consumed when receiving the remote inference result.

Local inference is therefore more energy-efficient when the combined cost of on-device processing and result transmission is lower than the cost of sending the original data and receiving the remote result. This comparison assumes that both approaches perform the same sensing task under comparable operating conditions. Energy costs common to both approaches, such as image acquisition, are excluded, while gateway and cloud processing energy are outside the device-side measurement boundary. The break-even point depends on factors such as data size, model complexity, communication conditions, and hardware characteristics. Therefore, the reported energy savings should be interpreted as specific to the experimental conditions in [50], rather than as a general advantage of local inference.

This observation also changes how energy efficiency should be interpreted. Minimizing the power consumption of an individual component does not necessarily maximize network lifetime or information yield. System-level studies of wireless sensor networks indicate that longer operation often depends on how energy consumption is distributed among nodes and functions rather than on minimizing local consumption alone [51]. A more relevant objective is therefore to maximize the amount of reliable information obtained from the available energy budget. Such a perspective requires performance indicators that connect measurement quality with the computational and communication resources required to obtain and deliver that information [48].

The preferred operating point is also unlikely to remain constant. Environmental conditions, signal strength, communication quality, workload, and available energy can vary substantially during real-world operation, particularly in environmental monitoring, wearable devices, mobile platforms, and industrial sensing systems [49]. A configuration that is efficient under one set of conditions may therefore become suboptimal under another. Adaptive sensing architectures address this problem by modifying parameters such as sampling frequency, sensing duration, processing complexity, transmission rate, or node activity according to the current operating state.

These dependencies make co-design particularly relevant. Rather than optimizing sensing materials, device architecture, embedded processing, communication, and power management sequentially, their interactions can be considered during the same design process. This allows improvements at one layer to be evaluated against the additional demands they impose on other layers and supports the identification of configurations that provide an appropriate balance between performance, energy use, and system autonomy [38].

Computational models introduce another layer of this design problem. High-fidelity approaches such as finite element simulations can provide detailed representations of sensor behavior, but their computational requirements may limit their use in repeated design exploration, real-time estimation, adaptive control, or other cross-layer optimization tasks. Reduced-order and equivalent models can substantially decrease computation time while retaining acceptable accuracy. Reported examples show that computation can be reduced by several orders of magnitude while maintaining errors within a few percent of high-fidelity simulations [52]. Model fidelity should therefore be selected according to the intended function of the model rather than maximized independently of computational cost.

Taken together, these considerations show that intelligent sensor design is fundamentally a multi-objective problem. Sensing performance, computational effort, communication demand, energy availability, and system autonomy are interdependent rather than independent design variables. This view is consistent with broader developments in complex engineering systems, where data acquisition, modeling, computation, and decision-making are increasingly integrated within a common operational framework [53].

On this basis, four system-level trade-offs are considered throughout this review: measurement accuracy versus energy consumption, latency versus energy savings, local computation versus communication load, and model fidelity versus computational efficiency.

In practical system design, these trade-offs can be expressed through a set of coupled design variables, objectives, and constraints. Key design variables include the sensing rate and resolution, model complexity, processing location, transmission frequency, transmitted data volume, and node duty cycle. The main objectives are to maintain sufficient sensing or inference quality while reducing energy consumption and latency and extending system lifetime. These objectives are constrained by residual node energy, available memory and computational capacity, communication bandwidth, and application-specific latency requirements. Increasing the sensing rate or resolution can improve measurement quality, but it also increases the amount of data that must be processed and transmitted. Similarly, a more complex local model may improve inference performance or reduce transmitted data, but at the cost of additional computational energy and latency. Offloading computation to an edge or cloud platform reduces the local processing burden but introduces communication energy and network delay. Residual energy therefore affects which operating configuration remains feasible over time and directly links short-term sensing, computation, and communication decisions to long-term system lifetime.

**Figure 1:**
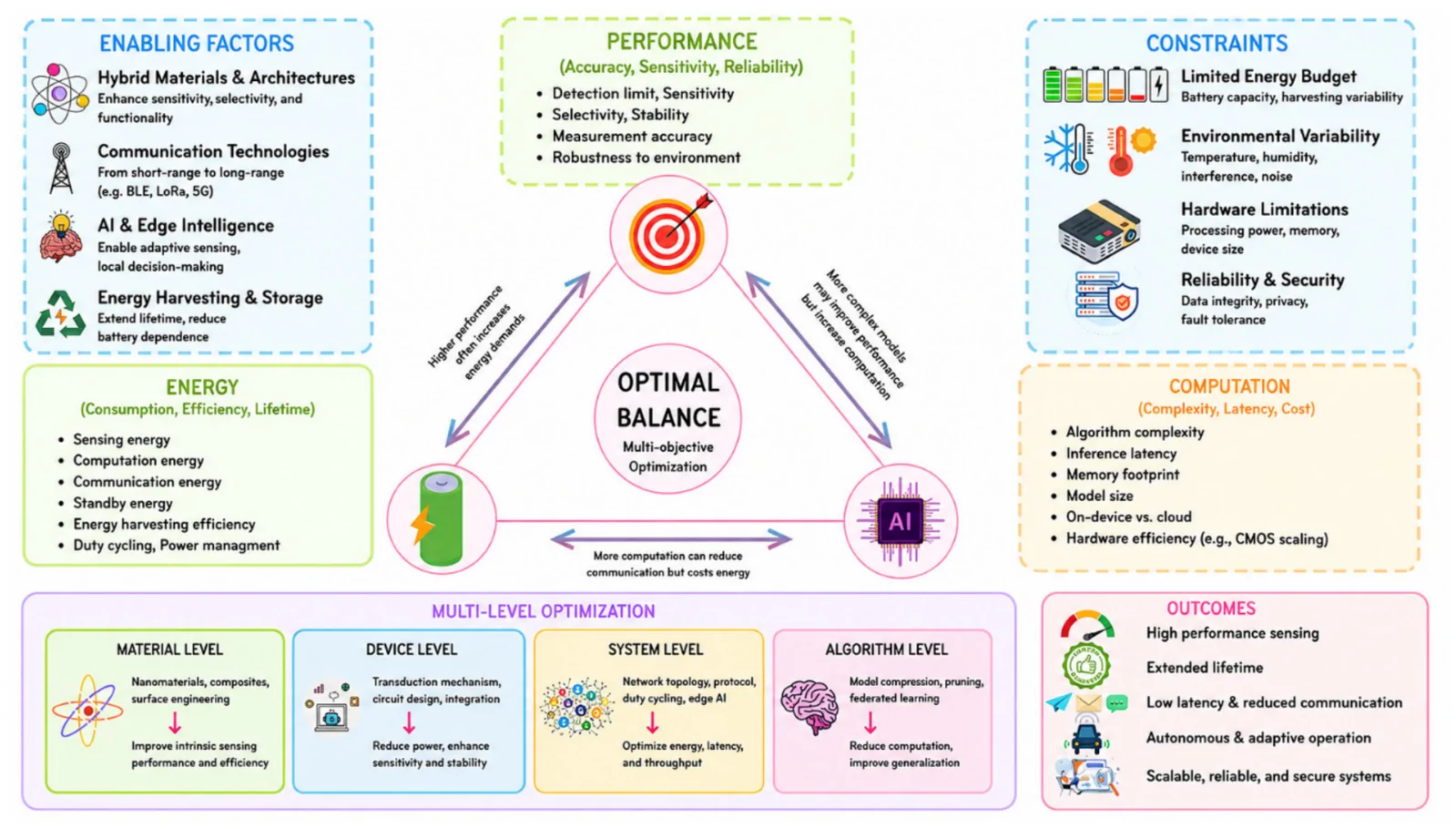
Conceptual system-level framework for energy-aware AI-enabled intelligent sensing systems. The framework shows how sensing performance, computational requirements, communication demand, and energy availability are interconnected and influence the overall operation of intelligent sensing systems.

Figure 1 summarizes the conceptual framework adopted in this review. Overall system performance is treated as an emergent property of interactions among sensing, data acquisition, computation and signal processing, communication, and energy management. The framework therefore provides a common basis for examining how design decisions at one layer propagate through the system and influence energy efficiency, information quality, and operational autonomy.

The framework can be applied as a practical design and evaluation tool by examining how a change in one system component affects the remaining resource dimensions. For example, consider an autonomous environmental monitoring node with a limited energy budget, local TinyML inference, and wireless communication. Increasing the sensing rate may improve temporal resolution, but it also increases the amount of data to be processed and transmitted. Local inference can reduce communication demand by filtering or summarizing the acquired data, although it introduces additional computation and memory use. A lower transmission rate can then reduce communication energy but may increase latency or reduce the amount of information available remotely. The framework therefore allows these design choices to be evaluated together rather than independently and helps identify which of the four system-level trade-offs becomes dominant under a given operating condition. Integrated analysis of the four key system-level trade-offs in energy-aware intelligent sensing systems are shown in Table 2.

The four trade-offs are closely related rather than independent. Increasing the sensing rate, for example, can improve measurement quality but also increases the amount of data that must be processed and transmitted. Local processing can reduce this communication load, although more complex inference increases computational energy and latency. Similarly, aggressive duty cycling can extend system lifetime while reducing responsiveness. The appropriate design therefore depends on the application requirements, available energy, communication conditions, and computational resources rather than on the optimization of any single metric.

## 3. Energy Constraints and Resource-Aware System Design

In distributed and autonomous environments, sensor nodes typically rely on limited energy sources, including batteries or energy harvested from ambient sources such as solar radiation, mechanical vibrations, or radio-frequency signals. Within the framework considered in this review, energy availability is treated not only as a physical constraint but also as a design variable that determines how sensing, computation, communication, and adaptive operation can be configured. Energy constraints therefore affect measurement performance, latency, reliability, and system autonomy and must be considered at both the node and network levels.

### 3.1. Sources and Characteristics of Energy Constraints

The nature of energy constraints depends strongly on the power source. Battery-powered systems are limited by finite storage capacity and therefore require careful management of the available energy budget to maintain long-term operation. Energy-harvesting systems can support extended autonomous operation, but the harvested power is often intermittent and varies with environmental conditions. Solar harvesting depends on illumination conditions, vibration-based harvesting requires mechanical activity, and radio-frequency energy harvesting is generally associated with low available power density.

System design must therefore account not only for average power consumption but also for temporal variations in energy availability, peak loads, storage capacity, and periods during which harvested energy is insufficient. These constraints become particularly important when sensing, processing, and communication loads change dynamically. The limitations can be severe in compact autonomous platforms. In miniature robotic systems, for example, the power remaining for sensing after actuation and control may be limited to approximately 10–30 mW, restricting the complexity of the sensing architecture that can be supported [54].

### 3.2. Energy Consumption Distribution in Sensor Nodes

At the node level, the available energy is shared mainly among sensing, computation, memory access, and communication. Their relative contributions depend on the sensing mechanism, processing architecture, communication technology, sampling strategy, and application requirements.

In many wireless sensor network architectures, communication represents a major component of the energy budget because radio transmission and reception must be performed repeatedly throughout operation. Reducing the amount or frequency of transmitted data can therefore provide substantial energy savings. Local computation can reduce communication demand by extracting features, compressing data, or making decisions directly at the node, but algorithm execution and memory access also consume energy. Moving processing from the communication link to the sensor node consequently does not guarantee a reduction in total energy consumption. The relevant balance depends on the energy required for local computation relative to that required for wireless transmission [55].

The sensing mechanism itself may also impose a substantial energy cost. Active sensing, high sampling rates, thermally activated transducers, and multimodal architectures can require continuous or periodic power input. Gas sensors illustrate this constraint clearly. Electrochemical gas sensors generally operate at low power and provide high sensitivity but can suffer from cross-sensitivity to interfering gases. Solid-state gas sensors can provide high sensitivity and rapid response, although their operating temperature often results in higher power consumption and their response may also be affected by interfering species. Microelectromechanical systems (MEMS) provide one route to reducing this demand. Miniaturized sensing structures and localized microheaters can maintain the required sensing temperature with lower energy expenditure while preserving compact dimensions and rapid response [56,57].

Piezoelectric devices represent a different approach because the sensing element can generate an electrical signal directly from mechanical deformation. This reduces or, for the sensing element itself, can eliminate the need for active excitation. Cylindrical arrays based on poly(vinylidene fluoride) (PVDF), for example, have been used to determine the direction of an applied force over a broad angular range from signals generated by longitudinal and circumferential sensing elements. The dependence of response amplitude on applied load also provides a basis for estimating force magnitude [58]. Such architectures are relevant when low-power operation must be combined with multidirectional mechanical sensing.

Passive or near-passive tuning mechanisms can also reduce continuous power demand. Liquid-metal elements have been used to tune resonant frequency without continuous external power, providing a possible route to lower-energy tunable microwave sensing systems [1].

For applications requiring long-term autonomy under variable operating conditions, combining energy harvesting, storage, and controlled energy distribution can provide greater robustness than relying exclusively on a single source. Hybrid power architectures allow the harvested energy, stored energy, and instantaneous load to be managed according to operating conditions [59]. This reflects the broader node-level constraint that every stage of sensing and signal processing contributes to the total energy budget [60].

Resource sharing provides another way to reduce unnecessary hardware activity. In Internet of Vehicles (IoV) systems, sensor virtualization allows a physical sensor to serve several application requests through virtual sensing instances. The IoV-Cloud architecture reported in [61] creates such virtual sensors on demand by dynamically selecting available vehicle sensors. Reinforcement learning (RL) was used to select sensors according to location, availability, and provisioning cost. Although energy consumption was not the primary optimization objective, shared use and adaptive selection of physical sensors can reduce redundant sensor activation and associated sensing, processing, and communication loads.

A more direct route to eliminating local battery demand is passive wireless sensing. In one reported architecture, an ultra-high frequency radio-frequency Identification (UHF RFID) tag was integrated with a capacitive sensing element and antenna. Changes in the measured parameter produced a phase shift in the radio-frequency signal transmitted by the RFID tag. Water level, flooding, and moisture sensing were demonstrated without a local battery or separate active measurement circuit [62]. Such systems are particularly relevant for distributed and difficult-to-access deployments where battery replacement is undesirable.

Communication performance introduces an additional energy constraint. For a general wireless sensor link, the signal-to-interference-plus-noise ratio (*SINR*) can be expressed as:

(1) $$\mathrm{SINR}=\frac{P_{s}}{P_{i}+P_{n}}$$

where *P_s_* is the received useful signal power, *P_i_* is the interference power, and *P_n_* is the noise power, all expressed in watts (*W*). The achievable data rate can be approximated as:

*R* = *Blog*_2_(1 + *SINR*) (2)

where *R* is the achievable data rate in bits per second (bit/s), and *B* is the channel bandwidth in hertz (Hz). The expression assumes an idealized Gaussian channel in which interference is treated as additional Gaussian noise and the *SINR* is constant over the considered bandwidth. The *SNR* considers only the desired signal power and noise power $\mathrm{SNR}=\frac{P_{s}}{P_{n}}$. When interference is negligible *P_i_* ≈ 0, *SINR* approaches to *SNR*. Equation (2) therefore represents an ideal capacity bound under these assumptions. Actual data rates may be lower due to fading, protocol overhead, and hardware limitations.

Energy efficiency can be expressed as:

(3) $$\mathrm{EE}=\frac{R}{P}$$

where *EE* is the communication energy efficiency, expressed in bits per joule (bit/J), *R* is the achievable data rate in bit/s, and *P* is the average power consumed by the communication subsystem in watts (W). In this formulation, *P* includes the power used for radio transmission or reception, depending on the evaluated communication operation, but excludes sensing, data processing, and AI inference energy. The resulting metric therefore describes communication-level energy efficiency rather than the energy efficiency of the complete sensing platform. These relationships illustrate how communication quality, achievable data rate, and radio power consumption are connected [63].

### 3.3. Resource-Aware Design Strategies

Energy limitations can be addressed at several levels of sensor architecture. One of the most established approaches is duty cycling, in which sensor nodes alternate between active and low-power states. This reduces average energy consumption when continuous measurement or communication is unnecessary, but the duration and timing of sleep intervals must be selected so that relevant events are not missed.

Adaptive sampling provides a related mechanism at the sensing level. Measurement frequency can be increased when the monitored signal varies rapidly and reduced during relatively stable periods. In this way, energy expenditure is linked to the information content of the measurement rather than to a fixed sampling schedule.

Data reduction can further decrease the communication load. Local preprocessing, feature extraction, compression, and event detection allow a node to transmit only selected information instead of raw measurements. Edge computing and embedded artificial intelligence extend this concept by performing inference directly at the sensing node. Their benefit depends on whether the reduction in communication energy compensates for the additional energy required for computation and memory access.

It is important to distinguish conventional energy-saving techniques from AI-based approaches. Duty cycling, fixed transmission schedules, threshold-based sampling, and standard data compression do not necessarily require AI. They can reduce energy consumption with relatively low computational overhead and are often sufficient when sensing conditions are predictable. AI-based approaches, in contrast, use learned models to interpret sensor data, detect patterns, predict changes, or adjust system operation. For example, machine learning can support event detection and adaptive sensing, while reinforcement learning can help select sensing or communication policies under changing conditions. However, these benefits come with additional processing, memory, and energy requirements. AI is therefore most useful when its ability to improve sensing decisions or reduce unnecessary operations outweighs the cost of running the model.

Hardware-level optimization includes low-power microcontrollers, optimized analog front ends, application-specific integrated circuits, improved transduction mechanisms, and reduced parasitic losses. Materials and device engineering can therefore contribute directly to the system energy budget rather than being considered independently from electronic architecture.

**Figure 2:**
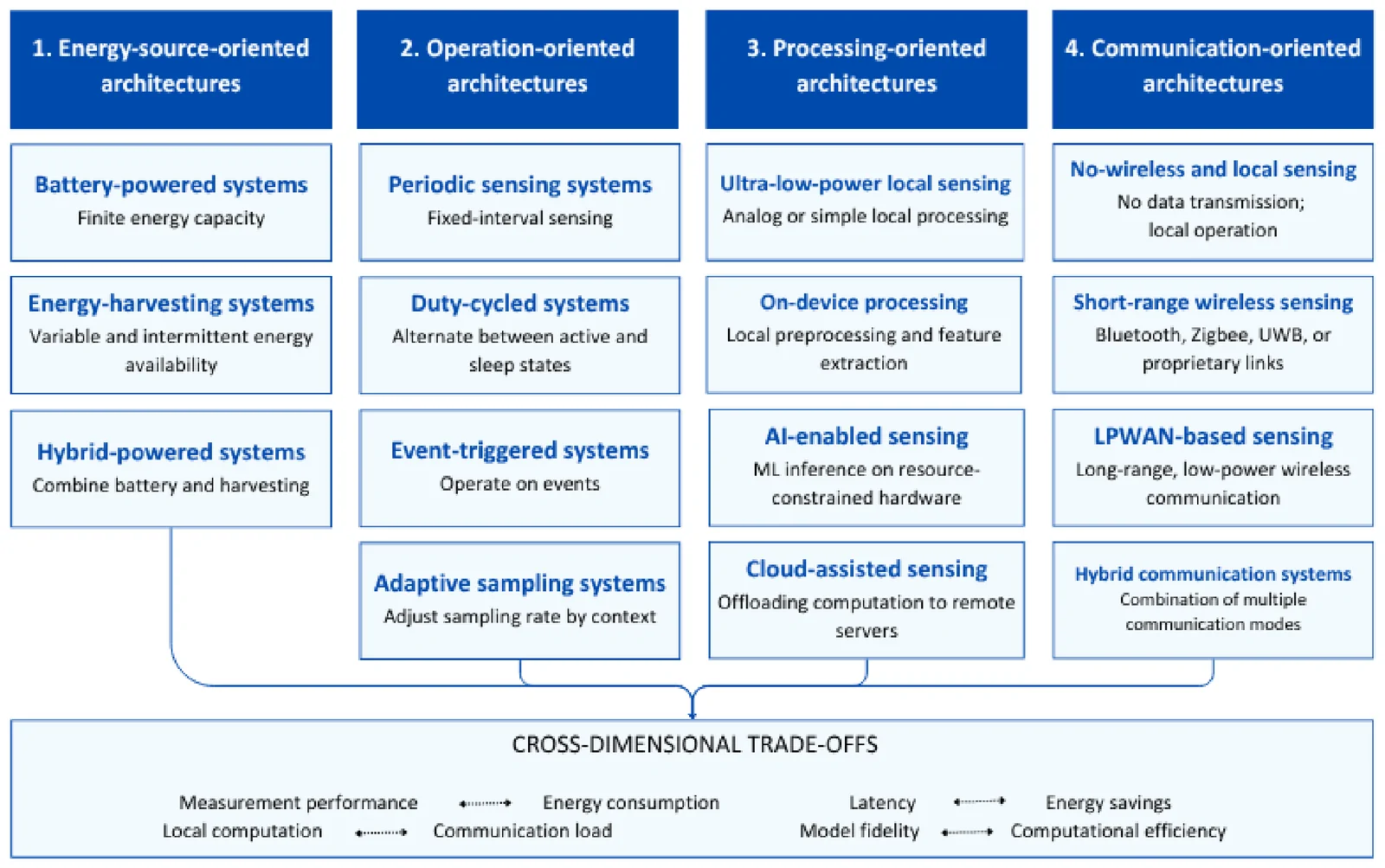
Conceptual taxonomy of power-aware sensor system architectures.

Co-optimization of sensing hardware and signal reconstruction provides another route to low-power operation. Systems combining passive sensing structures with power-aware reconstruction methods have been reported to operate at power levels of approximately 10 mW while retaining structural similarity values above 80% in reconstructed spatial information [54]. Figure 2 organizes these approaches according to four architectural dimensions: energy source, operational mode, processing model, and communication strategy.

The taxonomy groups sensing architectures into four dimensions based on energy source, operational mode, processing model, and communication strategy. Energy-source-oriented architectures differ in how energy is supplied and managed, while operation-oriented approaches focus on reducing consumption through periodic, duty-cycled, event-triggered, or adaptive operation. Processing-oriented architectures describe where and how sensor data are processed, whereas communication-oriented approaches focus on data transmission requirements and communication efficiency. These approaches are not mutually exclusive and can be combined within the same sensing system. The proposed architectural taxonomy is mapped to representative studies to highlight recurring design patterns, key architectural characteristics, and existing research gaps, as summarized in Table 3.

Table 3 shows how the four architectural dimensions are reflected in the reviewed studies. Approaches related to energy sources and operational modes mainly focus on managing available energy and controlling when sensors operate. In contrast, processing models and communication strategies address where data are processed and how they are transmitted. In practice, these approaches are often combined. For example, a sensor node powered by energy harvesting may use adaptive duty cycling, local inference, and low-power communication to reduce energy consumption while maintaining sensing performance.

The comparison also highlights several research gaps. Many studies examine energy efficiency, sensing performance, AI processing, or communication separately, rather than evaluating their combined effects under changing operating conditions. Direct comparisons between approaches are also difficult because studies use different hardware platforms, workloads, and evaluation metrics. In addition, more research is needed to understand how adaptive sensing systems perform over long periods when energy availability and network conditions vary. The proposed taxonomy helps organize these findings and provides a common basis for comparing architectural approaches and identifying their main trade-offs.

The increasing use of embedded AI adds computational load to this energy allocation problem. In addition to sensing and communication, AI-enabled nodes must support inference, memory access, and model execution. Their energy efficiency therefore depends on the balance among data quality, local processing, and communication rather than on processor efficiency alone. Table 4 shows that low-power sensor system differ not only in their absolute power consumption but also in the consequences of reducing that consumptions.

#### Application of the Proposed Framework to Representative Sensing Architectures

To illustrate how the proposed framework can be used in practice, three representative sensing architectures [64,66,70] were examined using the four dimensions of the taxonomy: energy source, operational mode, processing model, and communication strategy. These studies were selected because they represent different approaches to energy management, data processing, and communication, including both AI-enabled and conventional sensor systems.

The system in [64] combines solar energy harvesting, adaptive duty cycling, on-device TinyML inference, and LoRaWAN/BLE communication. This approach integrates several energy-saving strategies and reduces the need for frequent data transmission. However, its reported performance is based on simulation, leaving questions about long-term operation under real-world conditions.

In contrast, ref. [66] uses battery-powered sensor nodes with synchronized sleep cycles and gateway-based data analysis. This approach reduces energy consumption without requiring AI-based processing on the sensor node. However, the relatively long reporting interval limits responsiveness. Similarly, ref. [70] combines low-power operation with multi-hop communication for structural monitoring. While this architecture supports extended communication coverage, forwarding introduces additional communication overhead and delay.

Applying the framework to these studies shows that different architectural choices address different system constraints. For applications requiring frequent measurements and local decision-making, the combination of embedded inference and adaptive operation in [64] offers a potentially useful design approach. Where periodic measurements are sufficient, conventional duty cycling, as demonstrated in [66], may provide a simpler alternative. In applications requiring distributed monitoring over larger areas, multi-hop communication, as used in [70], may be more appropriate, although the associated energy and latency costs must be considered.

This comparison also highlights two research gaps. First, AI-enabled and conventional architectures are often evaluated under different conditions, making it difficult to determine when the additional computational cost of AI is justified. Second, the combined effects of energy availability, sensing frequency, processing location, and communication strategy are rarely evaluated using consistent system-level metrics. The proposed framework helps identify these limitations and provides a structured basis for selecting and evaluating sensing architectures according to application requirements.

Energy-harvesting architectures improve autonomy but remain dependent on variable energy input. Periodic and duty-cycled operating modes reduce average power at the cost of increased latency or possible event loss, whereas ultra-low-power local architectures may sacrifice remote accessibility and network integration. Power consumption alone is therefore insufficient for evaluating energy-aware sensing systems; measurement continuity, responsiveness, communication capability, and autonomy must also be considered.

These constraints can be grouped into three interconnected levels. At the power-source level, capacity, availability, and intermittency define the energy that can be supplied. At the node level, sensing, computation, and communication compete for that energy. At the network level, routing, scheduling, security, and distributed processing determine how efficiently the available resources are used.

### 3.4. Network-Level Trade-Offs and Multi-Objective Optimization

Network-level energy management introduces additional trade-offs because changes intended to reduce communication cost can affect latency, reliability, throughput, security, or network stability. For AI-enabled sensing systems, these network-level mechanisms are particularly relevant because they determine how much data can be transferred, where processing can be performed, and how much energy remains available for local or distributed inference.

Routing and clustering are widely used to distribute communication load among nodes. Cluster heads can aggregate or forward data on behalf of nearby nodes, thereby reducing redundant transmissions, but their selection must account for residual energy, communication distance, and local network conditions. Energy-aware cluster-head selection has been reported to improve both network lifetime and stability compared with conventional selection strategies [73].

The magnitude of the improvement depends strongly on the protocol and operating conditions. In the study reported in [74], the proposed power-aware approach produced substantially longer network lifetime and reduced average energy consumption relative to the compared conventional methods. Other multi-objective routing studies have reported energy savings of up to 70% under their optimized configurations [75]. These results should be interpreted in the context of the individual network architectures and simulation conditions rather than as universal performance gains.

A broader comparison of routing protocols shows why a single energy metric is insufficient. Protocol performance can depend simultaneously on energy consumption, delay, throughput, packet delivery ratio, scalability, and computational complexity [76]. Improvements in one metric may therefore be accompanied by deterioration in another. Composite evaluation methods have been proposed to rank algorithms across several criteria, although the resulting ranking remains dependent on the selected metrics and their weighting [76].

Physical network topology introduces another source of inefficiency. A logical routing step may correspond to several physical hops, increasing packet transmissions, energy consumption, and end-to-end delay. This effect is particularly important when communication represents a large fraction of the node energy budget [77].

Security requirements add further computational and communication load. Lightweight protocols can keep this overhead within the resource limits of embedded nodes; reported implementations have achieved computation times of approximately 4–5 ms, memory requirements below 10 KB, and communication overhead of a few hundred bytes per transaction [78]. Optimized secure-access protocols have also been reported with handshake delays below 30 ms and energy consumption of a few millijoules per authorization [78]. These examples illustrate that security must be included in the resource budget rather than treated as an independent layer.

The interaction between network efficiency and security is also evident in routing design. Reactive routing can reduce some resource requirements but may introduce security limitations, whereas more proactive mechanisms can increase computational and communication overhead [60]. The appropriate choice therefore depends on both threat requirements and the available energy budget.

Privacy introduces a related system-level constraint. Distributed sensing systems may collect sensitive data, while privacy-preserving mechanisms can require additional computation, memory, and communication. Approaches that limit the transfer of raw data can reduce exposure, but they may shift part of the processing burden to edge devices. Privacy should therefore be considered together with energy, latency, and computational constraints when selecting the system architecture.

Energy behavior can also be non-linear. Increasing the number of processing units or active resources may increase instantaneous power while reducing execution time, so the configuration with the lowest power does not necessarily produce the lowest total energy consumption [79]. Although this relationship has also been examined in larger computing systems, the same distinction between power and total energy is relevant when evaluating distributed sensing workloads.

Multi-parameter clustering provides a practical example of this type of optimization. In unmanned aerial vehicle (UAV)-assisted wireless sensor networks, cluster-head selection based on residual energy, communication distance, and antenna gain was reported to reduce energy consumption by up to 22.4% relative to low-energy adaptive clustering hierarchy (LEACH) and by 15.8% relative to hybrid energy-efficient distributed clustering (HEED). The same study reported longer network lifetime, higher packet delivery ratio, improved throughput, reduced delay, and fewer packet drops [80]. The result is relevant because optimization affects several system metrics simultaneously rather than energy consumption alone.

Application-specific physical constraints can further modify the optimization objective. In wireless body area networks, continuous communication can increase the temperature of sensor nodes and surrounding tissues. The study cited in [81] reported substantially different thermal behavior between conventional and thermally aware routing strategies. In such applications, routing decisions must account for thermal limits as well as energy consumption and communication performance.

Other clustering approaches incorporate residual energy, communication distance, node density, and previous cluster-head selection history. The mechanism reported in [82] increased network lifetime by approximately 4–8% and throughput by 16–24% relative to the compared LEACH-based approaches. The scale of improvement differs between studies, but the underlying design problem is the same: network operation must be optimized across several coupled objectives.

### 3.5. Toward Integrated Energy-Aware System Design

Energy-aware sensor design can therefore be formulated as a multi-objective problem in which sensing performance, computational effort, communication demand, reliability, and energy availability are optimized under changing operating conditions. The preferred configuration is application-dependent and may change during operation in response to variations in environmental conditions, workload, network quality, and available energy.

Distributed cyber-physical systems increasingly coordinate sensing, computation, and communication across edge and cloud resources. In such architectures, energy consumption becomes part of the operating policy rather than a fixed hardware characteristic. Real-time measurements and predictive models can be used to determine where data should be processed, how frequently sensors should operate, and when information should be transmitted [83].

Reinforcement learning provides one mechanism for adapting these decisions to changing resource availability. Sensor nodes can adjust sensing and transmission rates according to the available energy budget. The adaptive strategy reported in [67] increased the amount of transmitted data by up to 23% without depleting the battery, illustrating how operation can be adjusted to use available energy more effectively rather than simply minimizing activity.

The same principle applies when security is included in optimization. In cyber-physical sensor systems, clustering can reduce communication energy, while deep-learning-based intrusion detection can improve security at the cost of additional computation. Coordinated optimization of these functions has been reported to reduce energy consumption by more than a factor of two while maintaining detection accuracy of approximately 99% [84].

Energy-aware intelligent sensing should therefore be treated as a coordinated design problem spanning the power source, sensing mechanism, embedded computation, communication architecture, and operational policy. The achievable performance of the complete system depends on how energy is distributed among these functions and how the allocation changes in response to operating conditions. This system-level view provides the basis for the following sections, where hybrid sensing architectures and AI-based processing are examined in terms of both their functional benefits and their additional resource requirements [85].

Overall, the reviewed approaches show that no single energy-saving mechanism is consistently preferable across all sensing systems. Duty cycling, adaptive sampling, routing, and local processing can reduce energy consumption, but their benefits depend on workload, communication cost, energy availability, and latency requirements. More aggressive energy saving may reduce responsiveness or measurement continuity, while additional coordination can introduce computational and communication overhead. Direct comparison across studies also remains difficult because of differences in hardware, network configuration, and evaluation conditions.

## 4. Emerging Intelligent Sensing Architectures

### 4.1. Hybrid and Multimodal Sensing Architectures

Sensing technologies have evolved from single-function devices toward integrated systems capable of acquiring and processing multiple types of signals. In many modern platforms, sensing elements are combined with signal processing and communication modules, allowing measured signals to be processed locally or transferred to higher system levels for further analysis and control. Closed-loop architectures extend this functionality by using the acquired information to modify subsequent system operation, which is relevant to IoT platforms, wearable electronics, and human–machine interfaces [86].

In this context, hybrid sensing extends beyond the combination of different sensing materials. Hybrid architectures may integrate optical, electrical, electrochemical, mechanical, or other transduction mechanisms within the same device or system. Combining complementary modalities can increase the amount of information available for signal interpretation and, depending on the application, improve robustness under variable operating conditions [87].

The need for multimodal sensing is particularly evident in systems governed by several interacting physical processes. Mechanical, acoustic, optical, electrical, or electromagnetic signals may provide complementary information about the same monitored object or process. Their combined analysis can reduce ambiguity associated with a single measurement modality and improve performance under changing environmental or operating conditions [88].

Hybrid sensing architectures can be considered at three hierarchical levels: material- and sensing-level hybridization, device-level integration, and system-level integration. At the first level, different materials or transduction mechanisms are combined to improve the sensing response. Device-level integration combines several sensing functions within a common platform, whereas system-level integration links heterogeneous sensors with data processing, communication, and decision-making.

Advances in MXenes, carbon nanotubes, graphene derivatives, semiconductor heterostructures, and related materials have contributed to improvements in sensitivity, selectivity, and stability. Within the system-level scope of this review, however, these materials are considered enabling components whose practical value depends on their integration with processing electronics, communication, and energy management [89,90]. From an AI-enabled sensing perspective, sensor characteristics are relevant not only because they determine measurement quality, but also because they influence sampling requirements, data volume, preprocessing demand, and the computational workload presented to embedded AI models.

The examples in Table 5 show that the main advantage of hybrid and multimodal architectures lies in the complementary information provided by different sensing mechanisms. Their relevance therefore extends beyond improvements in the response of an individual sensor. At the system level, the additional information can support more reliable signal interpretation, wider measurement ranges, and operation under conditions in which a single sensing modality may be insufficient.

Hybrid sensing can also be implemented within distributed architectures. IoT-based systems can combine stationary sensor nodes, mobile sensing platforms, wireless communication, and remote processing to increase spatial coverage and support targeted measurements [98,100]. The resulting architecture allows different sensing resources to be used according to the measurement task rather than requiring every node to perform the same function continuously.

Multimodal data fusion provides a direct example of the benefit of complementary measurements. In the system reported in [99], the combination of inertial measurement unit (IMU) and visual data reduced the mean per-joint position error (MPJPE) to approximately 1.21 cm, compared with 15.21 cm for vision-only sensing and 5.48 cm for inertial-only sensing. The result demonstrates how fusion of independent sensing modalities can reduce uncertainty in position estimation.

These advantages are accompanied by additional resource requirements. Multiple sensing channels can increase data volume, processing requirements, synchronization complexity, and power consumption. Heterogeneous sensors may also operate at different sampling rates and produce data with different temporal and statistical characteristics. The benefit obtained from additional sensing modalities must therefore be considered against the computational, communication, and energy resources required to acquire and process their outputs.

**Figure 3:**
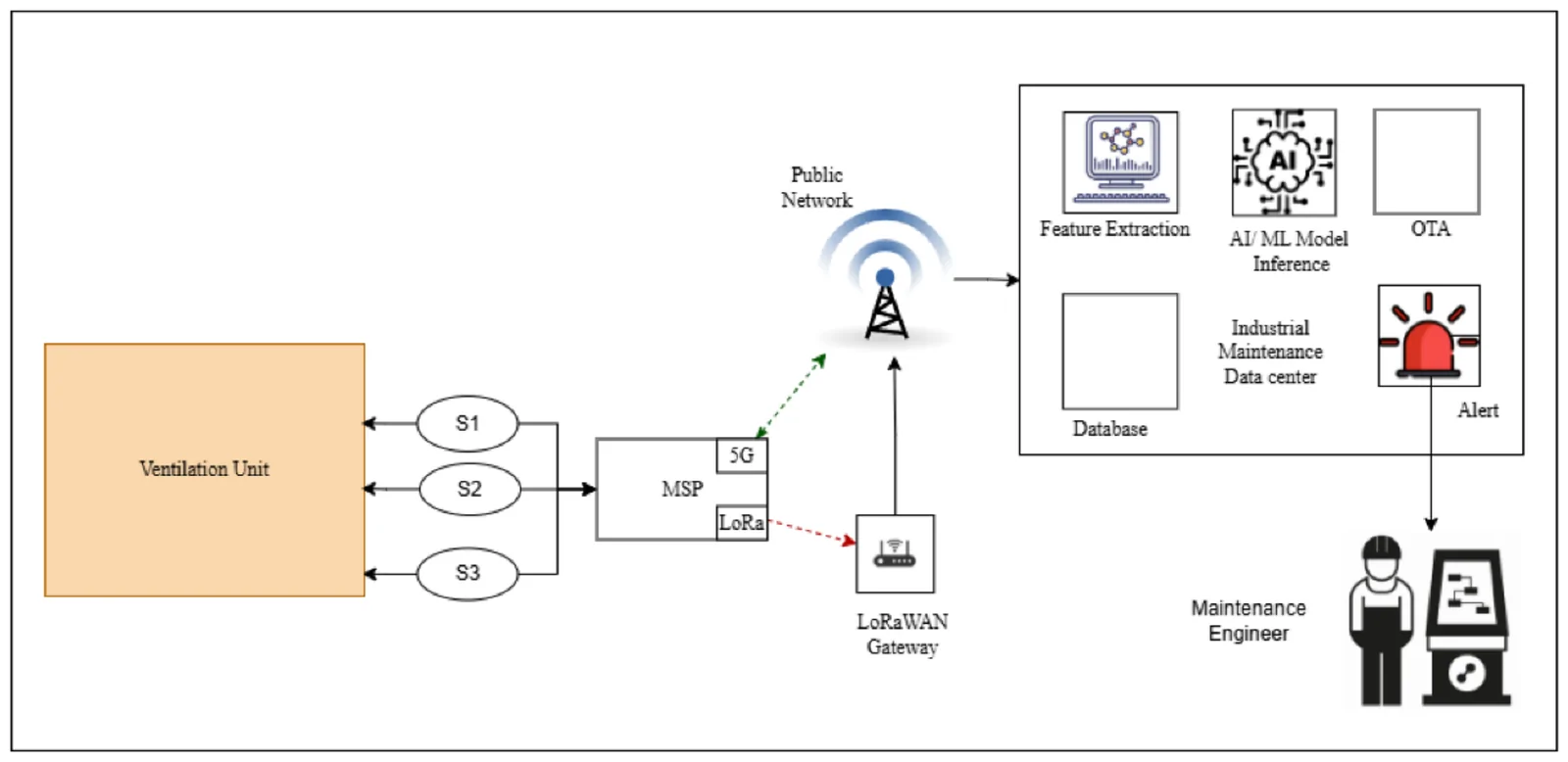
Hybrid LoRa–5G multi-sensor architecture for predictive maintenance, illustrating the integration of edge processing, low-power long-range communication, and high-bandwidth real-time data transmission pathways. Reproduced from [71].

At the network level, different communication technologies can also be combined according to data requirements. Comparative analyses of centralized, hierarchical, and distributed sensing systems indicate that architecture selection affects energy consumption, spatial coverage, and resilience [101]. In industrial IoT applications, hybrid communication based on low-power long-range communication (LoRa) and fifth-generation mobile networks (5G) provides one example of this approach [71]. Low-rate or condition-based information can be transmitted through LoRa, whereas larger or time-critical data streams can be transferred through 5G. The communication pathway can therefore be selected according to data volume, latency requirements, and available resources (Figure 3).

Hybrid platforms increasingly incorporate machine-learning methods for feature extraction, classification, and interpretation of multimodal measurements. Their use introduces an additional computational requirement, however, and the benefit of local intelligence must therefore be evaluated together with the associated processing, memory, communication, and energy costs.

Integrated structural designs provide another route to multimodal sensing by incorporating several transduction mechanisms within a single platform. Such designs can extend the measurable parameter range and provide complementary input signals for subsequent analysis [102]. Hybrid sensing should therefore be evaluated not only in terms of sensitivity or selectivity, but also in terms of the additional information obtained relative to the resources required to acquire and process it.

### 4.2. Distributed Intelligent Sensing Architectures

The use of hybrid and multimodal sensing increases both the volume and diversity of generated data. Processing all of this information at a central location can introduce communication and latency constraints, particularly in large-scale or resource-constrained sensor networks. Distributed architectures address this problem by allocating computational tasks among sensor nodes, edge devices, and cloud resources.

Conventional cloud-centric architectures transmit sensor data to remote servers for storage and processing. Cloud infrastructure provides substantially greater computational and storage capacity than embedded sensor nodes and is therefore suitable for complex analytics, long-term data management, and model training. Continuous transmission of raw measurements, however, increases communication traffic, latency, and the energy required for wireless data transfer [68].

**Figure 4:**
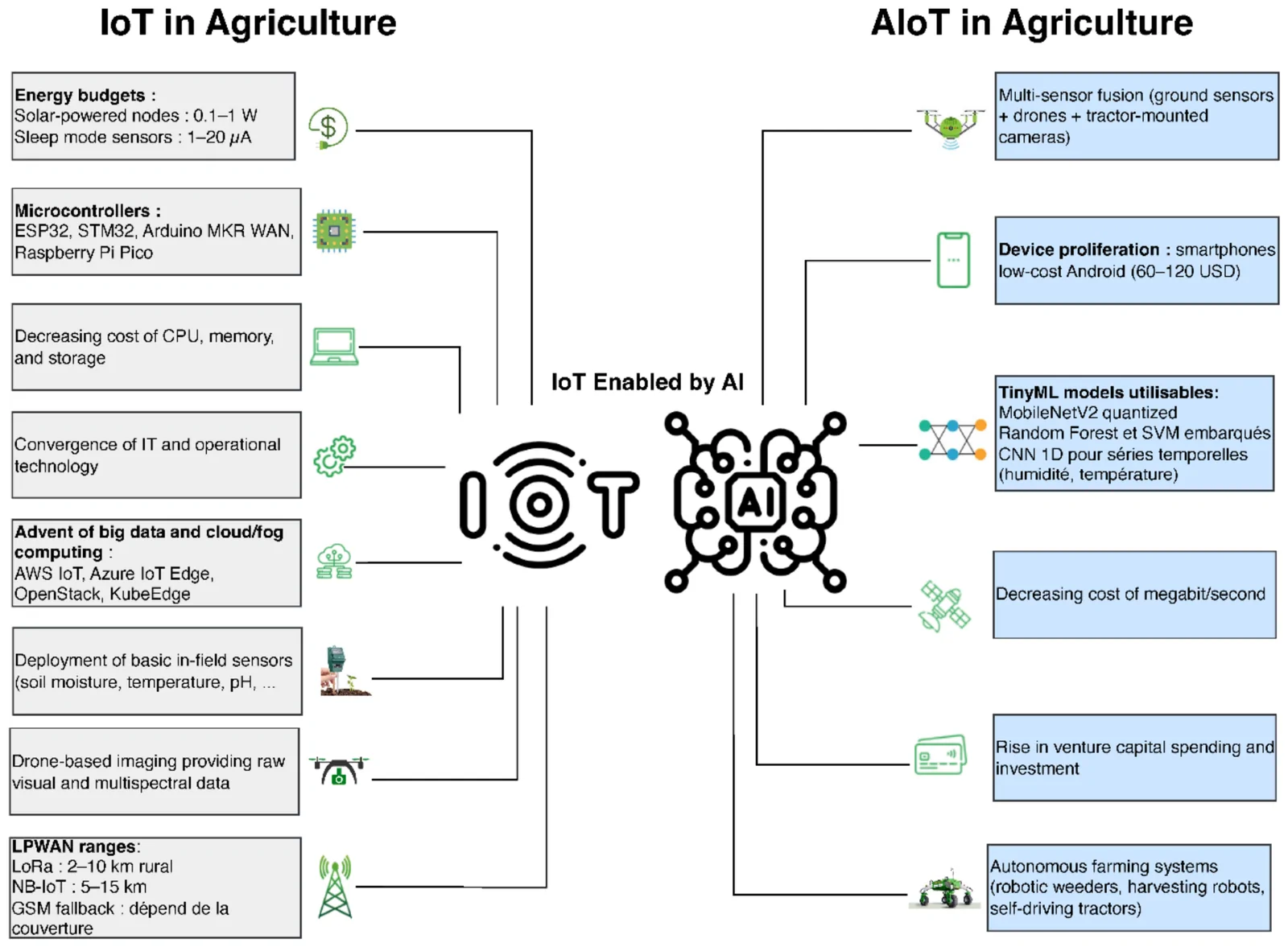
Conceptual illustration of the evolution from conventional cloud-centric IoT sensing systems toward AI-enabled sensing architectures under resource-constrained conditions, highlighting the transition toward distributed intelligence, edge computing, multimodal sensing, and energy-aware operation. Reproduced from [68].

Figure 4 illustrates the transition from centralized processing toward a distributed architecture. Although the original example is presented in an agricultural AIoT context, the underlying allocation of sensing, edge processing, and cloud resources is applicable to other distributed sensing systems.

In distributed architectures, computational tasks can be assigned according to their latency, resource, and data requirements. Sensor nodes may perform initial signal conditioning or simple event detection. Edge devices can support more demanding processing close to the data source, while cloud resources remain available for computationally intensive analysis, long-term storage, and model management. This division reduces the need to transfer all raw sensor data to remote servers.

The appropriate allocation depends on the application. Time-critical functions benefit from processing close to the sensor, whereas tasks requiring large computational or storage resources may remain at the edge or cloud level. Available energy, communication bandwidth, model complexity, and response-time requirements therefore influence where processing is performed [68]. In Table 6, Criteria for Computational Task Placement in Sensor-Edge–Cloud Architectures are presented.

Each processing approach has advantages and limitations. Local processing reduces the need to transmit raw sensor data and can provide faster responses, but it is limited by the memory, computing capacity, and energy available on the device. Edge processing allows more demanding tasks to be performed near the sensor, reducing its computational burden while avoiding some of the delays associated with cloud communication. However, it still depends on network connectivity and available edge resources. Cloud processing supports more complex models and large-scale data analysis, but transmitting sensor data to remote servers can increase communication energy and latency [68].

The most suitable approach depends on the application and the relative costs of computation and communication. For example, continuous monitoring with large data volumes may benefit from local filtering or inference, particularly when wireless transmission is energy-intensive. In contrast, applications requiring complex analysis or model training may benefit from edge or cloud resources, provided that communication costs and latency remain acceptable. Local processing is therefore not always the most energy-efficient option, especially when the computational workload is high or only small amounts of data need to be transmitted. Processing placement should be selected according to data volume, model complexity, network conditions, available energy, and application requirements.

Advances in low-power embedded processors, energy-efficient communication, and multimodal sensing have expanded the range of operations that can be executed near the point of measurement. Distributed sensing architectures consequently provide a means of adjusting the location of computation according to system constraints rather than assigning all processing permanently to either the sensor node or the cloud.

### 4.3. Embedded Intelligence for Modern Sensor Systems

Embedded intelligence refers to the execution of data-processing and decision functions directly within or close to the sensing platform. These functions can range from signal preprocessing and feature extraction to local inference and adaptive control. Their implementation is constrained by the processing capability, memory, and energy available at the embedded device [103].

Local processing can reduce the volume of information that must be transmitted through the network. Instead of continuously sending raw measurements, the sensor node can extract relevant features, identify events, or generate an inference result before communication. This can reduce bandwidth requirements and communication latency, although the resulting energy benefit depends on the computational and memory cost of the local processing itself [68,103].

Improved embedded hardware has made it possible to execute increasingly complex models on resource-constrained devices. Depending on the application, this allows sensor platforms to perform context-dependent processing, local classification, anomaly detection, or control without continuous dependence on cloud resources [104]. The extent to which these functions should be executed locally remains an architectural decision governed by model complexity, latency requirements, communication cost, and available energy.

The main functions of embedded intelligence considered in this review are summarized in Table 7.

TinyML provides an important implementation route for several of these functions. By reducing model size and computational requirements, TinyML enables inference on low-power microcontrollers that would otherwise be unable to execute conventional machine-learning models [68,105]. TinyML should therefore be considered an implementation approach for resource-constrained embedded intelligence rather than a separate sensing function.

Embedded intelligence does not remove the resource constraints discussed in the preceding sections. Local inference reduces communication only when useful information can be extracted before transmission, and increasingly complex models require additional computation, memory access, and energy. Its system-level value therefore depends on the balance between the information obtained locally and the resources required to produce that information.

The following section examines these computational constraints in greater detail, with particular emphasis on resource-aware artificial intelligence and the relationship between model complexity, inference performance, memory demand, and energy consumption.

Overall, hybrid sensing, distributed processing, and embedded intelligence can improve information quality and reduce reliance on centralized processing. However, they also increase data volume, synchronization needs, computational load, and energy consumption. Their practical benefit therefore depends on whether these added costs are compensated by better sensing performance or lower communication demand. A key remaining challenge is to adapt sensing and processing strategies as network conditions, workload, and available energy change.

## 5. Resource-Aware AI for Intelligent Sensor Systems

### 5.1. TinyML for Resource-Constrained Intelligent Sensing

The architectural evolution of sensing platforms has enabled AI to move from centralized cloud platforms toward embedded and edge devices operating under severe resource constraints. Consequently, modern AI algorithms must be designed not only to maximize prediction accuracy but also to minimize computational complexity, memory usage, inference latency, and energy consumption. Conventional convolutional neural networks, although highly accurate, are typically characterized by large model size and high computational complexity, which limits their applicability in energy-limited environments such as edge devices, wearable sensors, and distributed monitoring systems.

TinyML has become a practical approach for deploying machine-learning models on ultra-low-power microcontrollers and embedded sensing platforms. Techniques such as model compression, quantization, pruning, and hardware-aware optimization help reduce memory use, computational demand, and energy consumption while preserving acceptable inference performance. This makes TinyML well suited to battery-powered and energy-harvesting sensor systems, where both computing resources and communication budgets are limited. Performing inference locally can also reduce the amount of data that needs to be transmitted, although this shifts part of the energy and memory burden to the sensor node itself. Model selection should therefore consider not only model size, but also prediction performance, memory requirements, inference latency, communication demand, and overall energy consumption.

Recent reviews have examined software engineering, hardware–software co-design, optimization methods, and broader research trends in TinyML [43,44,106,107]. Resource-constrained implementations generally rely on compact models and model optimization techniques because microcontrollers provide substantially less memory, processing capability, and energy than conventional computing platforms [25]. Common model classes include compact convolutional neural networks, fully connected networks, decision trees, and autoencoders [25]. Their suitability depends on both the sensing modality and the target task. Convolutional networks, for example, can process image, audio, and time-series signals, whereas autoencoders are frequently used for anomaly detection when representative abnormal data are limited.

Model size alone does not adequately describe deployment requirements. Intermediate activations generated during inference may require more static random-access memory (SRAM) than the stored model weights [108,109]. A model with relatively few parameters can therefore still exceed the memory available on a microcontroller. Similarly, a reduction in the number of arithmetic operations does not necessarily produce lower latency if those operations are inefficiently supported by the processor or inference runtime. Model architecture and execution software must consequently be considered together.

MCUNet illustrates this hardware-aware approach by combining TinyNAS and TinyEngine. TinyNAS searches for neural-network architectures that satisfy device-specific memory, latency, and energy constraints, while TinyEngine is designed to reduce memory demand and improve execution efficiency on microcontrollers [108]. This approach differs from conventional model development in which the network is optimized independently and adapted to the target hardware only after training.

Depthwise separable convolution provides another common mechanism for reducing computational demand. MobileNetV2 uses depthwise convolutions together with inverted residual blocks to reduce the number of operations relative to conventional convolutional architectures [110]. Models originally designed for mobile processors, however, may still exceed the resources available on microcontrollers and may require further adaptation of network depth, width, or input resolution [108].

Neural architecture search can perform this adaptation systematically. MicroNets, for example, applies architecture search to resource-constrained image classification, keyword spotting, and anomaly-detection tasks [111]. Input resolution is also an important design variable because smaller inputs reduce the number of operations, the memory required for intermediate the activation and inference latency. Excessive reduction, however, can decrease prediction accuracy [108,109].

Peak SRAM consumption is particularly important in convolutional models because early network layers can generate large feature maps. MCUNetV2 addresses this limitation through patch-based inference, in which only portions of a feature map are processed at a given time, reducing peak memory requirements [109]. This demonstrates that resource optimization can involve the execution schedule as well as the neural-network architecture.

MCUNet, MCUNetV2, and MicroNets differ mainly in their deployment priorities. MCUNet combines neural architecture search with the TinyEngine runtime to reduce memory demand and inference latency, although the reported efficiency is closely tied to the co-designed execution engine. MCUNetV2 further lowers peak SRAM requirements through patch-based inference, but this approach can add computational and latency overhead when the execution strategy is not jointly optimized with the network architecture. MicroNets uses TensorFlow Lite Micro and targets commodity Cortex-M microcontrollers, which improves deployment portability but preserves framework and memory-management overhead. Overall, MCUNet focuses on hardware–software co-design, MCUNetV2 on peak-memory reduction, and MicroNets on portable deployment under explicit memory and latency constraints.

### 5.2. Hardware-Aware Model Evaluation

The performance of the same AI model can vary substantially across embedded platforms because execution depends on processor architecture, available memory, supported operators, and the inference library [112]. Evaluation on a desktop computer is therefore insufficient for determining whether a model is suitable for deployment on a sensor node.

Resource-aware evaluation should include prediction accuracy together with model size, peak SRAM usage, inference latency, and energy per inference. MLPerf Tiny provides benchmarking procedures based on accuracy, latency, and energy measurements for TinyML systems [113]. These metrics allow models to be compared in terms of deployability rather than prediction performance alone.

There is consequently no universally optimal lightweight model for intelligent sensing. Model selection depends on the input modality, required accuracy, available memory, inference frequency, communication strategy, and hardware support. In some cases, a slightly larger model may be preferable if its improved prediction quality reduces unnecessary sensing events or wireless transmissions. If the selected architecture still exceeds device limits, quantization, pruning, or other compression methods can be applied to reduce its memory and computational requirements.

Hardware operating conditions also affect the energy cost of inference. In complementary metal-oxide-semiconductor (CMOS) digital circuits, dynamic power depends on switching activity, capacitance, supply voltage, and operating frequency. Increasing computational throughput through higher clock frequencies can therefore increase power demand, particularly when higher frequencies require corresponding changes in operating voltage [114]. The energy required for inference is consequently determined not by clock frequency alone, but by the interaction among operating conditions, execution time, and computational workload.

At the algorithmic level, inference can be represented as a sequence of weighted operations and nonlinear transformations (Equation (4)). In simplified form:

(4) $$y=\sigma \left(\sum_{i=1}^{N}x_{i}w_{i}+b\right)$$

where *y* is the output of the neuron, *x_i_* is the *i*-th input, *w_i_* is the corresponding weight, *b* is the bias term, *N* is the number of inputs, and *σ*(∙) denotes the nonlinear activation function. Although individual operations can be efficiently implemented on embedded hardware, complete neural-network inference may involve a large number of such operations together with memory access and data movement. Training generally requires substantially greater computational and memory resources than inference and is therefore commonly performed outside highly constrained sensor nodes [25].

### 5.3. Information-Aware Resource Allocation

Resource-aware AI must also consider whether the information being processed or transmitted remains useful to the application. The urgency of information (UoI) provides one framework for incorporating both the freshness and task-dependent importance of sensed data. UoI-based formulations assign costs to outdated or inaccurate information, allowing sensing, computation, and communication resources to be allocated according to the current value of the information rather than solely according to its age [115].

This perspective is relevant to intelligent sensing because processing every measurement with the same model complexity or transmitting every observation at the same rate may use limited resources without providing a proportional improvement in decision quality. Task-dependent information value can therefore complement conventional measures such as latency, accuracy, and energy consumption when adaptive operation is required.

A related limitation of conventional model-selection procedures is the assumption that the model with the highest prediction accuracy is necessarily the most suitable for deployment. Hyperparameter optimization based exclusively on accuracy can produce models whose memory requirements or inference latency exceed the capabilities of embedded hardware [105]. Resource-aware optimization must therefore treat accuracy, computational cost, memory demand, and latency as simultaneous constraints.

### 5.4. Resource-Aware AI Within Distributed Sensor Architectures

Resource-aware AI operates within the broader distributed architectures discussed in the preceding section. In AI of Things (AIoT) systems, sensor nodes acquire data, embedded or edge devices perform time-sensitive preprocessing and inference, and cloud resources can support more computationally demanding analytics, model training, and long-term optimization [96]. The location of each computational task can therefore be selected according to latency, communication cost, available hardware resources, and energy constraints.

The role of AI in such systems is not limited to interpreting sensor measurements. AI models can also participate in adaptive resource allocation by influencing when sensing is performed, which data are processed locally, what information is transmitted, and when computational tasks are transferred to edge or cloud resources. Resource-aware AI therefore links model design with the sensing, computation, communication, and energy-management constraints of the complete platform [104].

The energy benefits of AI should also be considered in relation to conventional approaches. Simple threshold-based methods, fixed schedules, and rule-based control generally require fewer computational resources and may provide sufficient performance in stable environments. AI-based methods can offer greater flexibility by learning patterns from sensor data and adapting decisions to changing conditions. For example, local inference can reduce communication by transmitting only relevant results, while learning-based resource management can adjust sensing or transmission activity according to available energy and workload.

However, these advantages are not guaranteed. AI models introduce additional costs associated with computation, memory access, and, in some cases, model updates or training. A more complex model may improve prediction accuracy without producing a corresponding reduction in total energy consumption. The value of AI therefore depends on whether improvements in sensing quality, responsiveness, or resource allocation justify these additional costs. For applications with simple and predictable operating conditions, conventional techniques may remain the more practical and energy-efficient choice.

### 5.5. Distributed and Emerging AI Paradigms

Federated learning allows multiple edge or IoT devices to train a shared model without sending their raw data to a central server. This can improve privacy and reduce direct data sharing, but it also introduces new resource demands. Local training requires computation and memory, while frequent exchange of model updates can increase communication and energy consumption. Differences in device capabilities, unstable connections, and uneven energy availability can also affect participation. Its practical value therefore depends on the model size, update frequency, local workload, and network conditions.

Split computing distributes a neural network across the device and a more capable edge or cloud platform. The first part of the model runs locally, while intermediate features are sent for further processing. The choice of split point affects local computation, communication volume, latency, and energy use [116]. In some cases, intermediate feature maps may be larger than the original input and increase communication cost. A suitable split can instead reduce the processing burden on constrained devices. Dynamic partitioning can be useful when bandwidth and device resources change over time.

Neuromorphic computing offers another option for low-power edge intelligence [69]. Spiking neural networks and event-driven hardware process information only when events occur, which can reduce unnecessary computation and data movement. This is attractive for always-on and event-driven sensing applications that require low latency and low energy use. However, neuromorphic systems still face limitations related to training tools, hardware dependence, and cross-platform evaluation. Their benefits therefore depend strongly on the target application and hardware.

At the system level, the three approaches address different parts of the same resource-allocation problem. Federated learning balances local training against communication cost, split computing balances local computation against offloading and latency, and neuromorphic computing aims to reduce computation and memory-access energy.

Overall, more efficient AI models are not always the best choice at the system level. Compression, quantization, and hardware-aware optimization can reduce memory use, latency, and energy consumption, but may affect model performance or depend on particular hardware. Moving inference closer to the sensor can also reduce communication, but increases local processing demands. A key challenge is therefore to select model complexity and processing location according to the available hardware, workload, and energy budget.

## 6. Challenges and Future Directions

Significant progress has been made in intelligent and energy-efficient sensing systems, yet their deployment in practical applications still involves several unresolved challenges. One of the most persistent is the need to balance sensing performance, computational demand, and long-term energy autonomy, particularly in hybrid and energy-aware architectures [117,118]. These requirements are closely coupled, so improvements in one part of the system can impose additional demands on another.

Scalability presents a related challenge in distributed sensor networks. As the number of sensing nodes increases, coordination, communication, and resource allocation become more complex [119]. Clustering and adaptive routing can reduce communication overhead and improve energy efficiency, but they do not address all constraints associated with heterogeneous and dynamic sensing environments. Differences in sensor characteristics, changing operating conditions, calibration requirements, measurement reliability, and available energy can all influence network behavior and complicate resource management [120,121,122]. Further development of decentralized and self-organizing architectures is therefore important for large-scale deployments.

Resource-constrained AI introduces a different set of limitations. Edge-based inference can reduce the amount of data transmitted to remote servers, but it shifts part of the computational and energy burden to the sensor node. Model size, memory requirements, inference latency, and energy consumption must therefore be considered together. In addition, many deployed models are trained under relatively stable data distributions and may lose performance when sensing conditions change over time [123,124,125,126]. This creates a need for lightweight models that can adapt to non-stationary environments without imposing excessive computational or memory requirements.

Evaluation also remains highly application-specific. Sensor systems are often assessed using isolated metrics such as sensitivity, detection limit, latency, or energy consumption, which makes comparison across architectures difficult [127,128]. More informative evaluation frameworks should combine sensing quality with computational cost, communication demand, and energy efficiency. Such metrics would provide a more consistent basis for comparing different system designs and identifying appropriate operating trade-offs.

Explainability and trustworthiness become increasingly important as more decision-making is transferred to embedded AI. In autonomous sensing systems, reliable operation depends not only on prediction accuracy but also on robustness to changing conditions, transparent model behavior, and the ability to maintain performance over extended periods of operation. These requirements are particularly important when adaptive algorithms influence sensing, communication, or resource-allocation decisions.

Another unresolved issue is the transition from laboratory demonstrations to long-term deployment. Many sensing platforms are evaluated under controlled conditions, whereas practical operation involves changing temperature, humidity, interference, contamination, mechanical stress, and calibration drift [129,130,131]. Addressing these effects requires attention not only to sensing materials and device architecture but also to calibration stability, fault tolerance, maintenance requirements, and long-term resource management.

Future sensing platforms are therefore likely to rely on tighter coordination among sensing, embedded computation, communication, and energy management. Adaptive sampling, distributed inference, lightweight AI, and energy-aware control can allow system operation to change with workload, environmental conditions, and available resources. Low-power electronics, neuromorphic processing, and distributed AI may further expand the range of functions that can be performed locally, although their practical value will depend on measurable improvements in energy use, latency, reliability, and information quality.

These challenges reinforce the need for system-level design rather than independent optimization of individual components. The performance of an intelligent sensing platform ultimately depends on how sensing, computation, communication, and energy resources are balanced under the operating conditions of the intended application.

Several more specific research problems follow from the system-level framework considered in this review. First, sensing frequency and AI model complexity should be jointly adapted to changing energy availability rather than optimized separately. Future systems should determine when higher sampling or more complex inference provides enough additional information to justify its energy cost. Second, collaborative inference across sensor, edge, and cloud resources requires adaptive task placement based on residual device energy, network quality, latency requirements, and model complexity. Third, cross-platform evaluation remains difficult because energy and latency results are often reported for different hardware, workloads, and communication conditions. Common evaluation procedures are therefore needed to compare energy efficiency, inference performance, and system lifetime across heterogeneous platforms. Finally, long-term deployments require adaptive models and resource-management policies that remain effective under sensor drift, changing environmental conditions, and variable energy availability.

## 7. Conclusions

This review examines intelligent sensing systems from a system-level perspective, with particular attention to the interactions among sensing, computation, communication, and energy management. The analysis shows that these elements cannot be optimized independently, because changes in one part of the system affect resource use and performance elsewhere. This is reflected in the trade-offs between measurement accuracy and energy consumption, latency and energy savings, local computation and communication load, and model fidelity and computational efficiency.

An important distinction of the present review is that energy efficiency is examined in relation to the operation of the entire sensing system rather than through individual energy-saving techniques. Previous reviews have largely concentrated on wireless sensor network lifetime, energy harvesting, communication protocols, or the computational requirements of Edge AI and TinyML. Although these studies provide valuable insights into specific aspects of sensor operation, they do not always address the relationships between sensing performance, data processing, communication, and available energy resources. In this work, these relationships are examined through four recurring system-level trade-offs and a taxonomy based on energy source, operational mode, processing model, and communication strategy. This provides a common basis for comparing different sensing architectures and helps explain why improving the efficiency of an individual component does not necessarily reduce the energy consumption of the complete system.

Recent developments in hybrid and multimodal sensing, distributed cloud–edge architectures, embedded intelligence, and resource-aware AI provide several ways to manage these trade-offs. Moving computation closer to the sensing node can reduce communication demand and latency, while TinyML and other lightweight AI approaches make local inference more practical on constrained hardware. However, these benefits depend on memory availability, computational cost, communication requirements, and the energy budget of the complete system. The choice of processing architecture should therefore account for both the resources consumed by local computation and the potential savings achieved through reduced data transmission.

The analysis also reveals several limitations that should be considered when evaluating existing intelligent sensing platforms. Reported improvements in energy consumption, inference accuracy, or communication efficiency are often obtained under different operating conditions, making direct comparisons between systems difficult. In particular, results obtained from simulations or short-term laboratory experiments may not fully reflect the effects of sensor drift, changing environmental conditions, battery degradation, and variations in network quality during long-term operation. Future studies should therefore place greater emphasis on evaluating sensing quality, processing requirements, communication energy, and operational lifetime under comparable conditions. Such evaluations would help determine whether the advantages of embedded intelligence and adaptive resource management can be maintained outside controlled experimental environments.

Taken together, these findings indicate that sensing technologies, processing architectures, communication strategies, and energy resources should be considered jointly during system design. This is particularly important for distributed and autonomous platforms, where operating conditions and available resources may change over time. Future progress will depend not only on improvements in individual sensing or computing technologies but also on identifying operating configurations that provide an appropriate balance between information quality, responsiveness, computational demand, and long-term autonomy.

## Figures and Tables

**Table 1:** Comparison of the present review with representative surveys in closely related research areas.

Research Scope	Taxonomy/Framework	Energy Treatment	Edge–Cloud and AI Deployment	Cross-Layer Optimization	Open Challenges	Main Distinction from Present Review	Source
Energy efficiency in WSNs	Classification of energy-saving mechanisms	Strong focus on energy conservation, duty cycling, routing, and network lifetime	Limited	Includes network-level and cross-layer energy optimization	Lifetime, scalability, energy constraints	Focuses mainly on WSN energy efficiency rather than joint sensing-AI-communication-energy interactions	[39]
Energy sustainability in WSNs	Energy saving, harvesting, and transfer mechanisms	Strong focus on energy sustainability	Limited	Mainly node- and network-level energy management	Sustainable operation and energy availability	Does not explicitly integrate AI deployment, processing placement, and sensing-performance trade-offs	[40]
Energy-efficient and interoperable IoT systems	IoT-oriented classification of energy and interoperability approaches	Energy efficiency and harvesting	Includes fog computing and data analytics	Partial	Heterogeneity, interoperability, scalability	Broad IoT perspective without a unified sensing-computation-communication-energy trade-off framework	[41]
Edge AI architectures and applications	Edge-AI architecture and deployment perspective	Energy considered as a resource constraint	Strong focus on edge processing and AI task placement	Partial	Resource limitations, latency, scalability, deployment	Focuses primarily on AI computation and placement rather than sensing and energy-source interactions	[42]
TinyML at the extreme edge	TinyML workflows, reformability, tools, and benchmarking	Low-power MCU operation	Mainly on-device AI	Limited	Adaptability, resource constraints, deployment	Model- and device-oriented; communication and sensing-energy allocation are not jointly analyzed	[43]
TinyML technologies and deployment	Classification of models, hardware, tools, and applications	Energy-efficient embedded operation	Mainly on-device AI	Limited	Hardware limitations, portability, deployment	Focuses on TinyML optimization rather than cross-layer sensing-communication-energy interactions	[44]
Energy-aware AI-enabled intelligent sensing systems	Four-dimensional taxonomy: energy source, operational mode, processing model, communication strategy	Energy source, allocation, autonomy, and dynamic resource constraints	Sensor-edge–cloud task placement, TinyML, distributed AI, split computing	Explicitly links sensing, computation, communication, and energy through four recurring trade-offs	Scalability, dynamic resource allocation, cross-platform evaluation, trustworthy AI, long-term deployment	Provides a unified system-level framework connecting architectural choices with cross-layer trade-offs	Present review

**Table 2:** Consolidated analysis of the four system-level trade-offs in energy-aware intelligent sensing systems.

System-Level Trade-Off	Most Relevant Conditions	Major Influencing Factors	Representative Optimization Approaches	System-Design Implication
Measurement accuracy vs. energy consumption	High-rate, high-resolution, continuous, or multimodal sensing under limited energy availability	Sampling rate, sensing duration, measurement resolution, number of sensing modalities, sensor power demand	Adaptive sampling, duty cycling, event-triggered sensing, low-power sensing hardware	Higher measurement quality generally requires more sensing, processing, and transmission energy; the preferred operating point depends on the information required by the application
Latency vs. energy savings	Duty-cycled nodes, event-driven systems, intermittent communication, and delay-sensitive applications	Sleep interval, wake-up frequency, transmission schedule, network quality, processing location	Adaptive duty cycling, event-triggered wake-up, edge processing, adaptive scheduling	Longer sleep periods and less frequent communication reduce energy use but can increase response delay or lead to missed events
Local computation vs. communication load	Distributed sensing systems where raw-data transmission is energy-intensive or bandwidth-limited	Data volume, model complexity, memory access, radio energy, bandwidth, link quality	Local preprocessing, feature extraction, compression, TinyML inference, split computing	Local processing is advantageous when the energy saved by reducing communication exceeds the additional computation and memory cost
Model fidelity vs. computational efficiency	Embedded AI and resource-constrained modeling where memory, latency, or energy is limited	Model size, numerical precision, activation memory, computational complexity, hardware support, required prediction accuracy	Quantization, pruning, neural architecture search, hardware-aware optimization, reduced-order modeling	Higher model fidelity can improve prediction quality but increases memory, latency, and energy demand; model complexity should therefore match the target task and hardware

**Table 3:** Mapping of the proposed architectural taxonomy to representative studies, recurring design patterns, and research gaps.

Taxonomy Dimension	Recurring Patterns and Differences	Research Gaps	Source
Energy source	Battery-powered architectures prioritize finite energy budgets; harvesting-assisted systems combine ambient energy and storage to extend autonomy	Limited joint evaluation of variable energy availability, AI workloads, and sensing continuity	[40,64,65]
Operational mode	Periodic sensing and duty cycling reduce average energy demand; adaptive approaches adjust system activity according to operating conditions	Insufficient analysis of responsiveness, information loss, and sensing quality under adaptive energy management	[64,66,67]
Processing model	On-device AI reduces raw-data transfer; edge/cloud processing provides greater computing capacity; distributed processing balances computation and communication	Lack of consistent criteria for comparing processing placement under combined accuracy, energy, memory, and latency constraints	[42,43,44,64,68,69]
Communication strategy	Low-power and multi-hop communication prioritize energy and coverage; hybrid communication supports different bandwidth and latency requirements	Limited joint evaluation of communication selection, local AI processing, and total system energy consumption	[66,70,71]

**Table 4:** Contextual summary of representative resource-aware sensing architectures and source-reported operating characteristics.

Platform and Processing	Energy Strategy/Source	Communication	Evaluation Setting and Test Conditions	Source-Reported Power/Energy Characteristics	Latency/Timing	Reported Performance	Main Limitation/Trade-Off	Source
Cortex-M55 and Ethos-U55; on-device INT8 TinyML inference	Solar harvesting and Li-ion battery; adaptive duty cycling	LoRaWAN and BLE	MATLAB/Simulink R2024b system-level simulation; 24 h cycle; model-size and sampling-rate sweeps	2.90 mW mean; 6.1 µJ/inference	0.87 ms inference	94.2% accuracy; F1 = 0.91; 88% fewer transmissions	Simulation only; physical sensor warm-up and long-term hardware effects not experimentally validated	[64]
ATmega328P MCU; local sensing with gateway-based storage and analysis	Battery powered; sensor power gating; synchronized sleep cycle	XBee/DigiMesh, 900 MHz	Field deployment with 20 nodes over 1 km^2^; 20 min duty cycle	0.66 mW sleep; 515.46 mW peak TX; estimated 91-day battery life	~100 ms transmission burst	Packet delivery ≥ 95%	High TX peak power; long reporting interval reduces responsiveness	[66]
0.18 µm CMOS ASIC; local capacitance-to-digital conversion	Low-power circuit design; 2.0 V supply	No wireless communication	Laboratory hardware prototype; FPGA/DAQ test bench; 250 kHz clock	37.2 µW active; 4.47 pJ/step	0.8 ms per conversion	12.7-bit ENOB; 84.2 dB SNDR	Component-level device; parasitic capacitance and capacitor mismatch affect accuracy	[72]
TZ1041 and CC1310 MCUs; ADXL355; local acquisition with multi-hop forwarding	Battery operation with aggressive sleep modes	IEEE 802.15.4 g/e sub-GHz multi-hop	Laboratory power tests and Manhattan Bridge field deployment	0.97 mW non-sensing sleep; 33.43 mW TX; 32.14 mW RX	100 ms TDMA slot	>1 km range/hop; modal frequencies within ~5–6% of reference values	Multi-hop forwarding adds delay; concentrator requires external power	[70]
MEMS micro-hotplate with Pt heater and Pd-doped SnO_2_ sensing layer	Low-thermal-mass microheater	No wireless communication	Laboratory gas-chamber testing; ethanol 1–10 ppm; ~400 °C operating temperature	39 mW heating power	NR	Sensitivity 29.7 at 10 ppm; 1 ppm detection limit	Continuous heater power; component-level sensor requires external conditioning	[57,65]
dsPIC33 MCU; ADXL345 accelerometer; local FFT processing	Solar harvesting and Li-Po battery; duty cycling	BLE	Laboratory tests and 3-month field deployment on transmission lines in Manitoba	2.6 mW sleep; 41.1 mW acquisition/TX; 8.1 mW cycle average	30 s wake/acquisition cycle	Vibration range 5–50 Hz; field operation down to −20 °C	Limited BLE range; battery performance degrades under severe cold	[57,65]

NR: not reported or not applicable at the evaluated system level. Note: Power and energy values are reported as provided in the cited studies and refer to different system boundaries, operating modes, duty cycles, hardware platforms, workloads, and evaluation conditions. They are included for contextual characterization and are not intended for direct numerical comparison.

**Table 5:** Representative hybrid and multimodal sensing architectures and their contribution to intelligent sensor systems.

Architecture Type	Representative Technology	Main System Benefit	Contribution to Intelligent Sensing	Ref.
Hybrid material sensing	MXene- and carbon-based nanocomposite sensors	High sensitivity and signal stability	Provides high-quality input signals for subsequent processing and analysis	[91,92]
Dual-mode sensing	Capacitive and Resistive Sensing	Wide dynamic range	Enables robust multimodal data acquisition under varying operating conditions	[93]
Multimodal physiological sensing	Strain and temperature sensing	Simultaneous monitoring of multiple physiological parameters	Supports adaptive health monitoring and context-aware sensing	[94,95]
Hybrid gas sensing	Hybrid chemiresistive materials	High selectivity	Enables reliable environmental monitoring and intelligent gas detection	[96,97]
Distributed multimodal sensing	IoT multi-sensor networks	Large-scale distributed monitoring	Supports edge intelligence, sensor fusion, and adaptive decision-making	[71,98]
Vision-IMU fusion	Multimodal data fusion	Improved localization and perception accuracy	Reduces uncertainty in multimodal pose estimation	[99]

**Table 6:** Criteria for computation placement in sensor-edge–cloud architectures.

Processing Location	Favorable Conditions	Energy Implications	Latency and Network Considerations	Typical Tasks
Sensor node	Small models, limited data volume, strict response-time requirements, sufficient residual energy	Avoids raw-data transmission but consumes local computation and memory energy	Lowest communication latency; less dependent on network availability	Filtering, feature extraction, event detection, lightweight TinyML inference
Edge device	Moderate model complexity, nearby edge resources, constrained sensor energy, low-to-moderate latency requirements	Reduces processing burden on the sensor while avoiding repeated cloud transmission	Depends on local link quality; usually lower latency than cloud processing	Aggregation, intermediate inference, multimodal fusion, collaborative processing
Cloud server	Large models, high computational demand, non-critical latency, reliable connectivity	Minimizes local computation but may increase communication energy because more data must be transmitted	Highest dependence on bandwidth and network delay	Model training, large-scale analytics, long-term storage, complex inference

**Table 7:** Architectural role of embedded intelligence in modern intelligent sensor systems.

Embedded Intelligence Function	Architectural Role	Primary System Benefit	Representative References
Local inference	Executes AI models directly on embedded devices	Reduces communication latency and bandwidth requirements	[68,103]
Feature extraction	Processes raw sensor signals before transmission	Decreases communication load and improves data efficiency	[103,104]
Adaptive sensing	Dynamically adjusts sensing and processing according to operating conditions	Improves energy efficiency and system autonomy	[68,104]
Edge decision-making	Enables immediate local responses without cloud dependency	Supports real-time operation and reduced latency	[68,104]
Distributed AIoT integration	Coordinates sensing, edge computing, and cloud intelligence	Improves scalability, reliability, and resource utilization	[104]

## Data Availability

No new data were created or analyzed in this study.

## References

[1] Juan C., Grenier K., Zarifi M., Ebrahimi A., Martin F. (2025). Planar Microwave Sensors: State of the Art and Applications. IEEE Access.

[2] Wang P., Liu Q. (2011). Physical Sensors and Measurement. Biomedical Sensors and Measurement.

[3] Zong B., Wu S., Yang Y., Li Q., Tao T., Mao S. (2024). Smart Gas Sensors: Recent Developments and Future Prospective. Nano-Micro Lett..

[4] Lee J., Cho K., Kim J. (2024). Age of Flexible Electronics: Emerging Trends in Soft Multifunctional Sensors. Adv. Mater..

[5] Tang Y., Li Y., Chen P., Zhong S., Yang Y. (2025). Nucleic Acid Aptamer-Based Sensors for Bacteria Detection: A Review. Bioessays.

[6] Mansoor S., Iqbal S., Popescu S., Kim S., Chung Y., Baek J. (2025). Integration of smart sensors and IOT in precision agriculture: Trends, challenges and future prospectives. Front. Plant Sci..

[7] Chen S., Fan S., Qiao Z., Wu Z., Lin B., Li Z., Riegler M., Wong M., Opheim A., Korostynska O. (2025). Transforming Healthcare: Intelligent Wearable Sensors Empowered by Smart Materials and Artificial Intelligence. Adv. Mater..

[8] Kim S., Tentzeris M., Georgiadis A. (2019). Hybrid Printed Energy Harvesting Technology for Self-Sustainable Autonomous Sensor Application. Sensors.

[9] Timoshen A., Savochkin A. Using Electromagnetic Radiation to Power Autonomous Sensors. Proceedings of the 29th International Crimean Conference: Microwave & Telecommunication Technology (CRIMICO’. 2019).

[10] Sardini E., Serpelloni M. (2009). Passive and Self-Powered Autonomous Sensors for Remote Measurements. Sensors.

[11] Weddell A., Merrett G., Harris N., Al-Hashimi B. (2008). Energy harvesting and management for wireless autonomous sensors. Meas. Control.

[12] Arachchige K., Branch P., But J. (2023). Evaluation of Blockchain Networks’ Scalability Limitations in Low-Powered Internet of Things (IoT) Sensor Networks. Future Internet.

[13] Liu H., Wang Y., Yu J., Wang S., Chen H. (2025). Design and Implementation of an Ultra-Low-Power Hazardous Gas Monitoring System. Sensors.

[14] Park K., Kim S. (2020). Low-power column counter with a logical-shift algorithm for CMOS image sensors. Electron. Lett..

[15] Ullah S., Shen B., Islam S., Khan P., Saleem S., Kwak K. (2010). A Study of MAC Protocols for WBANs. Sensors.

[16] Zheng C., Yong C., Wei Q., Qiao F. (2025). Flexible Wearable Heart Rate Monitoring System and Low-Power Design: A Review. Sensors.

[17] Botta A., de Donato W., Persico V., Pescapé A. (2016). Integration of Cloud computing and Internet of Things: A survey. Future Gener. Comput. Syst.-Int. J. Escience.

[18] Wang T., Zhang G., Bhuiyan M., Liu A., Jia W., Xie M. (2020). A novel trust mechanism based on Fog Computing in Sensor-Cloud System. Future Gener. Comput. Syst.-Int. J. Escience.

[19] Khan B., Khan W., Masrur M., Khalid R., Awais M., Khan B., Khoo B., Abdullah S. (2025). Hybrid sensor integration in wearable devices for improved cardiovascular health monitoring. J. Sci.-Adv. Mater. Devices.

[20] Weng Z., Guan R., Zou F., Zhou P., Liao Y., Su Z., Huang L., Liu F. (2020). A highly sensitive polydopamine@hybrid carbon nanofillers based nanocomposite sensor for acquiring high-frequency ultrasonic waves. Carbon.

[21] Orlanducci S. (2018). Gold-Decorated Nanodiamonds: Powerful Multifunctional Materials for Sensing, Imaging, Diagnostics, and Therapy. Eur. J. Inorg. Chem..

[22] Shi W., Cao J., Zhang Q., Li Y., Xu L. (2016). Edge Computing: Vision and Challenges. IEEE Internet Things J..

[23] Zhou Z., Chen X., Li E., Zeng L., Luo K., Zhang J. (2019). Edge Intelligence: Paving the Last Mile of Artificial Intelligence with Edge Computing. Proc. IEEE.

[24] Ray P. (2022). A review on TinyML: State-of-the-art and prospects. J. King Saud Univ.-Comput. Inf. Sci..

[25] Saha S., Sandha S., Srivastava M. (2022). Machine Learning for Microcontroller-Class Hardware: A Review. IEEE Sens. J..

[26] Schizas N., Karras A., Karras C., Sioutas S. (2022). TinyML for Ultra-Low Power AI and Large Scale IoT Deployments: A Systematic Review. Future Internet.

[27] Antonini M., Pincheira M., Vecchio M., Antonelli F. (2023). An Adaptable and Unsupervised TinyML Anomaly Detection System for Extreme Industrial Environments. Sensors.

[28] Özer E., Iskefiyeli M., Azimjonov J. (2021). Toward lightweight intrusion detection systems using the optimal and efficient feature pairs of the Bot-IoT 2018 dataset. Int. J. Distrib. Sens. Netw..

[29] Baciu V., Cause J., Morillo A., Garcia-Naranjo J., Stiens J., da Silva B. (2023). Anomaly Detection in Multi-Wavelength Photoplethysmography Using Lightweight Machine Learning Algorithms. Sensors.

[30] Ho H., Nguyen T., Huy D., Nguyen L. (2025). Bio-Inspired Algorithms in NLP Techniques: Challenges, Limitations and Its Applications. CMC-Comput. Mater. Contin..

[31] Chahine K. (2024). Machine Learning in Active Power Filters: Advantages, Limitations, and Future Directions. AI.

[32] Chen Y., Cheng Y., Jie Y., Cao X., Wang N., Wan Z. (2019). Energy harvesting and wireless power transmission by a hybridized electromagnetic-triboelectric nanogenerator. Energy Environ. Sci..

[33] Kim K., Cottone F., Goyal S., Punch J. (2010). Energy Scavenging for Energy Efficiency in Networks and Applications. Bell Labs Tech. J..

[34] Wu J., Mo Z., Gao X., Xin W., Shi W., Park J. (2025). Artificial intelligence assisted wearable flexible sensors for sports: Research progress in technology integration and application. Int. J. Smart Nano Mater..

[35] Wang C., He T., Zhou H., Zhang Z., Lee C. (2023). Artificial intelligence enhanced sensors-enabling technologies to next-generation healthcare and biomedical platform. Bioelectron. Med..

[36] Belabbaci N., Anaadumba R., Alam M. (2025). Recent Advancements in Wearable Hydration-Monitoring Technologies: Scoping Review of Sensors, Trends, and Future Directions. JMIR Mhealth Uhealth.

[37] Trigona C., Salerno G., Fromm R., Schott L., Kanoun O., Derbel F., IEEE Study of Energy Consumption and Performance in an Autonomous Sensor Node with On-Demand Communication and Wake-Up Receiver as a Function of Computational Complexity. Proceedings of the 2025 IEEE International Instrumentation and Measurement Technology Conference, I2MTC.

[38] Zieni B., Ritchie M., Mandalari A., Boem F. (2025). An Interdisciplinary Overview on Ambient Assisted Living Systems for Health Monitoring at Home: Trade-Offs and Challenges. Sensors.

[39] Rault T., Bouabdallah A., Challal Y. (2014). Energy efficiency in wireless sensor networks: A top-down survey. Comput. Netw..

[40] Evangelakos E., Kandris D., Rountos D., Tselikis G., Anastasiadis E. (2022). Energy Sustainability in Wireless Sensor Networks: An Analytical Survey. J. Low Power Electron. Appl..

[41] Rana B., Singh Y., Singh P. (2021). A systematic survey on internet of things: Energy efficiency and interoperability perspective. Trans. Emerg. Telecommun. Technol..

[42] Velaga K., Guo Y., Yu W. (2025). Edge AI for Smart Cities: Foundations, Challenges, and Opportunities. Smart Cities.

[43] Rajapakse V., Karunanayake I., Ahmed N. (2023). Intelligence at the Extreme Edge: A Survey on Reformable TinyML. ACM Comput. Surv..

[44] Tsoukas V., Gkogkidis A., Boumpa E., Kakarountas A. (2024). A Review on the emerging technology of TinyML. ACM Comput. Surv..

[45] Kerdabadi M., Ghazizadeh R., Farrokhi H., Najimi M. (2023). Joint Sensing Times Detection Thresholds and Users Association Optimization in Multi-Channel Multi-Antenna Cognitive Radio Networks. Int. J. Eng..

[46] Wagner J., Winger H., Cherif C., Ellinger F. (2023). Smart Glove With Fully Integrated Textile Sensors and Wireless Sensor Frontend for the Tactile Internet. IEEE Sens. Lett..

[47] Wang Q., Xu Z., Yang L. (2024). Collaborative Charging Scheduling in Wireless Charging Sensor Networks. CMC-Comput. Mater. Contin..

[48] Sapsanis C., Sophocleous M., Andreou A., Georgiou J. (2022). Trade-Offs in Sensor Systems Design: A Tutorial. IEEE Sens. J..

[49] Lai J., Ye B., Mihailidis A. (2026). Anomaly Detection Technologies for Dementia Care: Monitoring Goals, Sensor Applications, and Trade-offs in Home-Based Solutions—A Narrative Review. J. Appl. Gerontol..

[50] Sabovic A., Aernouts M., Subotic D., Fontaine J., De Poorter E., Famaey J. (2023). Towards energy-aware tinyML on battery-less IoT devices. Internet Things.

[51] Zhang W., Zhang Z., Chao H., Liu Y., Zhang P. (2017). System-Level Energy Balance for Maximizing Network Lifetime in WSNs. IEEE Access.

[52] Yuan C., Schütz A., Hohlfeld D., Bechtold T. (2023). Towards System-Level Simulation of a Miniature Electromagnetic Energy Harvester Model. Electronics.

[53] Chalgham W., Wu K., Mosleh A. (2020). System-level prognosis and health monitoring modeling framework and software implementation for gas pipeline system integrity management. J. Nat. Gas Sci. Eng..

[54] Bai Y., Garg N., Roy N. (2026). SPiDR: Microstructure-Assisted Vision for Ubiquitous Tiny Robots. Commun. ACM.

[55] Szymczyk M., Augustyniak P. (2022). Selected Energy Consumption Aspects of Sensor Data Transmission in Distributed Multi-Microcontroller Embedded Systems. Electronics.

[56] Wang H., Morando S., Gaillard A., Hissel D. (2021). Sensor development and optimization for a proton exchange membrane fuel cell system in automotive applications. J. Power Sources.

[57] Chen Y., Li M., Yan W., Zhuang X., Ng K., Cheng X. (2021). Sensitive and Low-Power Metal Oxide Gas Sensors with a Low-Cost Microelectromechanical Heater. ACS Omega.

[58] Lee Y., Neubauer J., Kim K., Cha Y. (2020). Multidirectional Cylindrical Piezoelectric Force Sensor: Design and Experimental Validation. Sensors.

[59] Knight C., Davidson J., Behrens S. (2008). Energy Options for Wireless Sensor Nodes. Sensors.

[60] Yang R., Patil M., Narayana P., Jayaram D., Keerthi K., Sudhakaryadav N., Chithaluru P., Kumar S., Elminaam D., Alsekait D. (2024). EDSSR: A secure and power-aware opportunistic routing scheme for WSNs. Sci. Rep..

[61] Abbes S., Rekhis S. (2024). Reinforcement Learning for Intelligent Sensor Virtualization and Provisioning in Internet of Vehicles (IoV). IEEE Access.

[62] Khalid N., Mirzavand R., Saghlatoon H., Honari M., Mousavi P. (2020). A Three-Port Zero-Power RFID Sensor Architecture for IoT Applications. IEEE Access.

[63] Enahoro S., Ekpo S., Al-Yasir Y., Uko M. (2025). Leveraging Reconfigurable Massive MIMO Antenna Arrays for Enhanced Wireless Connectivity in Biomedical IoT Applications. Sensors.

[64] Reis M. (2026). Low-Power Embedded Sensor Node for Real-Time Environmental Monitoring with On-Board Machine-Learning Inference. Sensors.

[65] Zanelli F., Castelli-Dezza F., Tarsitano D., Mauri M., Bacci M., Diana G. (2021). Design and Field Validation of a Low Power Wireless Sensor Node for Structural Health Monitoring. Sensors.

[66] Wu F., Tan C., Sarvi M., Rüdiger C., Yuce M., IEEE Design and Implementation of a Low-Power Wireless Sensor Network Platform Based on XBee. Proceedings of the 2017 IEEE 85th Vehicular Technology Conference (VTC Spring).

[67] Habeeb F., Szydlo T., Kowalski L., Noor A., Thakker D., Morgan G., Ranjan R. (2022). Dynamic Data Streams for Time-Critical IoT Systems in Energy-Aware IoT Devices Using Reinforcement Learning. Sensors.

[68] Kasereka S., Mbayandjambe A., Bazie I., Zeufack H., Ocama O., Hassan E., Kyamakya K., Tashev T. (2026). From IoT to AIoT: Evolving Agricultural Systems Through Intelligent Connectivity in Low-Income Countries. Future Internet.

[69] Anand K., Bhargav V.N. (2022). Split computing: DNN inference partition with load balancing in IoT-edge platform for beyond 5G. Meas. Sens..

[70] Tronci E., Nagabuko S., Hieda H., Feng M. (2022). Long-Range Low-Power Multi-Hop Wireless Sensor Network for Monitoring the Vibration Response of Long-Span Bridges. Sensors.

[71] Mohanram P., Schmitt R. (2025). Hybrid Long-Range-5G Multi-Sensor Platform for Predictive Maintenance for Ventilation Systems. Electronics.

[72] Wei R., Wang W., Xiao X., Chen Q. (2019). A Low-Power Delta-Sigma Capacitance-to-Digital Converter for Capacitive Sensors. IEEE Access.

[73] Houssein E., Saad M., Çelik E., Hu G., Ali A., Shaban H. (2024). An enhanced sea-horse optimizer for solving global problems and cluster head selection in wireless sensor networks. Clust. Comput.-J. Netw. Softw. Tools Appl..

[74] Elyyan R., Darabkh K. (2025). Power-aware clustering and routing protocol utilizing virtual hexagonal cells and computational intelligence for smart cities with dual mobility and power-constrained IoT sensors. Results Eng..

[75] Ibrahim A., Hashim F., Sali A., Noordin N., Fadul S. (2022). A Multi-Objective Routing Mechanism for Energy Management Optimization in SDN Multi-Control Architecture. IEEE Access.

[76] Lonkar B., Karmore S. (2022). Statistical Evaluation of Power-Aware Routing Protocols for Wireless Networks: An Empirical Study. Int. J. Intell. Inf. Technol..

[77] Cheklat L., Amad M., Omar M., Boukerram A. (2021). CHEARP: Chord-based Hierarchical Energy-Aware Routing Protocol for Wireless Sensor Networks. Comput. Sci. Inf. Syst..

[78] Chen L., Wang W., Huang J., Lu J., Guo R. (2026). AI-Enhanced Lightweight Key Agreement and Secure Access for Power Wireless Sensor Networks. Secur. Priv..

[79] Artioli M., Borghesi A., Chinnici M., Ciampolini A., Colonna M., De Chiara D., Loreti D. (2025). C6EnPLS: A High-Performance Computing Job Dataset for the Analysis of Linear Solvers’ Power Consumption. Future Internet.

[80] Paul P., Pushpa T. (2025). Design of energy aware crop monitoring clustering mechanism for UAV -based sensor networks by using aperture antenna. Discov. Comput..

[81] Shahbazi Z., Byun Y. (2020). Towards a Secure Thermal-Energy Aware Routing Protocol in Wireless Body Area Network Based on Blockchain Technology. Sensors.

[82] Sennan S., Kirubasri A.Y., Pandey D., Alghamdi S. (2022). EACR-LEACH: Energy-Aware Cluster-based Routing Protocol for WSN Based IoT. CMC-Comput. Mater. Contin..

[83] Cicceri G., Tricomi G., D’Agati L., Longo F., Merlino G., Puliafito A. (2023). A Deep Learning-Driven Self-Conscious Distributed Cyber-Physical System for Renewable Energy Communities. Sensors.

[84] Alajmi M., Nour M., Hassine S., Alkhonaini M., Hamza M., Yaseen I., Zamani A., Rizwanullah M. (2022). Energy Aware Secure Cyber-Physical Systems with Clustered Wireless Sensor Networks. CMC-Comput. Mater. Contin..

[85] Kanoun O., Bradai S., Khriji S., Bouattour G., El Houssaini D., Ben Ammar M., Naifar S., Bouhamed A., Derbel F., Viehweger C. (2021). Energy-Aware System Design for Autonomous Wireless Sensor Nodes: A Comprehensive Review. Sensors.

[86] Xu K., Lu Y., Takei K. (2021). Flexible Hybrid Sensor Systems with Feedback Functions. Adv. Funct. Mater..

[87] Butt M. (2026). Thin-Film Sensors for Industry 4.0: Photonic, Functional, and Hybrid Photonic-Functional Approaches to Industrial Monitoring. Coatings.

[88] Hassan M., Kirwin R., Rakurty C., Mahajan A. (2026). Advanced Sensor Technologies in Cutting Applications: A Review. Sensors.

[89] Tripathi M., Deokar G., Casanova-Chafer J., Jin J., Sierra-Castillo A., Ogilvie S., Lee F., Iyengar S., Biswas A., Haye E. (2024). Vertical heterostructure of graphite-MoS2 for gas sensing. Nanoscale Horiz..

[90] Shahat M., Khamees A., Ghitas A., Ezzat H. (2026). Highly sensitive hierarchically structured Si-based UV sensor-photodetectors via optimized ZnO-Al2O3 nanocomposite architectures. Sci. Rep..

[91] Chen J., Song T., Wang X., Zhou Y., Wang T., Zhang X., Zhao Y., Yang B., Zhang Y., Chen J. (2024). Ultrasensitive and wide-range MXene/PDMS piezoresistive sensors inspired by rose petals. Nano Energy.

[92] Li S., Fu Q., Pan C. (2022). A multi-functional wearable sensor based on carbon nanomaterials reinforced TPU fiber with high sensitivity. J. Alloys Compd..

[93] Ping Y., Li K., Yuan C., Guo D., Yang Y. (2026). A Dual-Mode Flexible Sensor with Capacitive-Resistive Hybrid Response for Bolt Loosening Monitoring. Sensors.

[94] Zhang Y., Li Y., Du A., Wu Y., Zeng J. (2024). Layer-by-layer assembly of chitosan and carbon nanotube on cotton fabric for strain and temperature sensing. J. Mater. Sci. Technol..

[95] Wu H., Chai S., Zhu L., Li Y., Zhong Y., Li P., Fu Y., Ma L., Yun C., Chen F. (2024). Wearable fiber-based visual strain sensors with high sensitivity and excellent cyclic stability for health monitoring and thermal management. Nano Energy.

[96] Li Z., Chen J., Chen L., Guo M., Wu Y., Wei Y., Wang J., Wang X. (2020). Hollow Au/Polypyrrole Capsules to Form Porous and Neural Network-Like Nanofibrous Film for Wearable, Super-Rapid, and Ultrasensitive NH3 Sensor at Room Temperature. ACS Appl. Mater. Interfaces.

[97] Keshtkar S., Rashidi A., Kooti M., Askarieh M., Pourhashem S., Ghasemy E., Izadi N. (2018). A novel highly sensitive and selective H2S gas sensor at low temperatures based on SnO2 quantum dots-C60 nanohybrid: Experimental and theory study. Talanta.

[98] Habib M., Chukwuemeka C. (2025). Development of IoT-Based Hybrid Autonomous Networked Robots. Technologies.

[99] Xefteris V., Syropoulou A., Pistola T., Kasnesis P., Poulios I., Tsanousa A., Symeonidis S., Diplaris S., Goulianas K., Chatzimisios P. (2025). Multimodal fusion of inertial sensors and single RGB camera data for 3D human pose estimation based on a hybrid LSTM-Random forest fusion network. Internet Things.

[100] Sunggat M., Alua A., Palvan K., Meruert N. (2026). Internet of Things for the Visually Impaired: Design of a Wearable Obstacle-Detection and Navigation Device. J. Emerg. Technol. Comput. (JETC).

[101] Li H., Shafieezadeh M. (2025). Smart sensor architecture selection for coastal marine monitoring. Appl. Water Sci..

[102] Wang Y., Yoshida J., Takeda Y., Yoshida A., Kaneko T., Sekine T., Kumaki D., Tokito S. (2024). Printed Composite Film with Microporous/Micropyramid Hybrid Conductive Architecture for Multifunctional Flexible Force Sensors. Nanomaterials.

[103] Shalaginov A., Azad M. (2021). Securing Resource-Constrained IoT Nodes: Towards Intelligent Microcontroller-Based Attack Detection in Distributed Smart Applications. Future Internet.

[104] Bitam T., Yahiaoui A., Boubiche D., Martínez-Peláez R., Toral-Cruz H., Velarde-Alvarado P. (2025). Artificial Intelligence of Things for Next-Generation Predictive Maintenance. Sensors.

[105] Preuveneers D., Tsingenopoulos I., Joosen W. (2020). Resource Usage and Performance Trade-offs for Machine Learning Models in Smart Environments. Sensors.

[106] Lakshman S., Eisty N.A.C.M. Software Engineering Approaches for TinyML based IoT Embedded Vision: A Systematic Literature Review. Proceedings of the 2022 IEEE/ACM 4th International Workshop on Software Engineering Research and Practices for the IOT (SERP4IOT).

[107] Han H., Trimi S., Lee S. (2026). Tiny Machine Learning (TinyML): Research trends and future application opportunities. Array.

[108] Lin J., Chen W., Lin Y., Cohn J., Gan C., Han S. MCUNet: Tiny Deep Learning on IoT Devices. Proceedings of the 34th International Conference on Neural Information Processing Systems.

[109] Lin J., Chen W., Cai H., Gan C., Han S. MCUNetV2: Memory-Efficient Patch-based Inference for Tiny Deep Learning. Proceedings of the 35th International Conference on Neural Information Processing Systems.

[110] Sandler M., Howard A., Zhu M., Zhmoginov A., Chen L., IEEE MobileNetV2: Inverted Residuals and Linear Bottlenecks. Proceedings of the 2018 IEEE/CVF Conference on Computer Vision and Pattern Recognition (CVPR).

[111] Banbury C.Z.C., Fedorov I., Matas R., Thakker U.G.D., Reddi V.J., Mattina M., Whatmough P.N. (2021). MicroNets: Neural network architectures for deploying TinyML applications on commodity microcontrollers. Proc. Mach. Learn. Syst..

[112] David R., Duke J., Jain A., Reddi V.J., Jeffries N., Li J., Kreeger N., Nappier I., Natraj M., Regev S. (2021). TensorFlow Lite Micro: Embedded machine learning for TinyML systems. Proc. Mach. Learn. Syst..

[113] Banbury C., Reddi R.V., Torelli P., Holleman J., Jeffries N., Kiraly C., Montino P., Kanter D., Ahmed S., Pau D. MLPerf Tiny benchmark. Proceedings of the Neural Information Processing Systems Track on Datasets and Benchmarks.

[114] Xiang Z., Zheng Y., He M., Shi L., Wang D., Deng S., Zheng Z. (2022). Energy-effective artificial internet-of-things application deployment in edge-cloud systems. Peer-To-Peer Netw. Appl..

[115] Ju Z., Rafiee P., Ozel O. (2022). Optimizing Urgency of Information through Resource Constrained Joint Sensing and Transmission. Entropy.

[116] Li H., Ge L., Tian L. (2024). Survey: Federated learning data security and privacy-preserving in edge-Internet of Things. Artif. Intell. Rev..

[117] Lonkar B., Charde P., Kuthe A., Dehankar A., Dandekar P., Puri N., Banabakode D. (2025). A review of hybrid clustering and routing protocols for the hotspot problem in wireless sensor networks. Peer-To-Peer Netw. Appl..

[118] Rana A., Kaur K., Kaur P., Bhatti E. (2025). Energy-efficient protocols for environmental monitoring in wireless sensor networks: A review. J. Ambient Intell. Smart Environ..

[119] Arellanes D., Lau K. (2020). Evaluating IoT service composition mechanisms for the scalability of IoT systems. Future Gener. Comput. Syst.-Int. J. Escience.

[120] Pidy L., Ngossaha J., Karunathilake T., Tsakou S., Förster A. (2025). An Efficient Cluster-Based Routing Protocol for Enhanced Data Delivery and Stability in VANETs: Cluster-Based Ant-Colony Routing With Adaptive Cluster Head. IET Netw..

[121] Patil A., Balasubramanyam A., Ryu J., Chakravarthi B., Chai Y. (2021). An Open-Source Platform for Human Pose Estimation and Tracking Using a Heterogeneous Multi-Sensor System. Sensors.

[122] Kou F., Liu Y., Li C., Qin H., Liu Y. (2026). AI Methods in Sensor Calibration. Sensors.

[123] Petri I., Chirila I., Gomes H., Bifet A., Rana O. (2022). Resource-Aware Edge-Based Stream Analytics. IEEE Internet Comput..

[124] Sharobiddinov D., Siddiqui H., Saleem A., Mezquita G., Vargas D., Díez I. (2025). Edge-Based Autonomous Fire and Smoke Detection Using MobileNetV2. Sensors.

[125] Anandhi D., Valli S. (2018). An algorithm for multi-sensor image fusion using maximum a posteriori and nonsubsampled contourlet transform. Comput. Electr. Eng..

[126] Wang J., Chen L., Al Faruque M., IEEE DOMINO: Domain-Invariant Hyperdimensional Classification for Multi-Sensor Time Series Data. Proceedings of the 2023 IEEE/ACM International Conference on Computer Aided Design, ICCAD.

[127] Wang D., Liu F., Gao Y., Yin H., Wang L. (2025). Novel Metrics and Wavelet Attention GRU for Gas Mixture Recognition With Reduced Sensors. IEEE Trans. Ind. Inform..

[128] Cong Q., Oh S., Fan W., Luo S., Althoefer K., Zhang D. (2026). TacEva: A Performance Evaluation Framework for Vision-Based Tactile Sensors. Adv. Intell. Syst..

[129] Mo X., Zhang L., Wang H., Luo B., Zhang J., Cai C., Nie S. (2026). High-Performance Hydrogels for Triboelectric Tactile Sensing: Principles, Design, and Applications. Adv. Funct. Mater..

[130] Yin W., Liu Z., Zhang S., Harlukowicz R., Ikram Q., Niu C., Xue Y. (2026). SensWorkflow: A high-performance framework for remote sensing big data processing on heterogeneous clusters. Big Earth Data.

[131] Yu Y., Liao X., Feng W. (2025). Recent development of elastomer-based smart sensing materials and structures. Adv. Compos. Hybrid. Mater..

